# High-Temperature Corrosion in Biomass-Fired Systems: Mechanisms, Mitigation, Strategies, and Challenges for the Brazilian Bioenergy Sector

**DOI:** 10.1021/acsomega.6c05615

**Published:** 2026-10-29

**Authors:** Allyson R. Simões, Moacir L. D. Junges, Rou-Yi Pan, Jonathan Utzig, Henry F. Meier, Rodrigo K. Decker, Vinicyus R. Wiggers

**Affiliations:** † 37874 Regional University of Blumenau (FURB), Chemical Engineering Department, Rua São Paulo, 3250, Blumenau, Santa Catarina 89030−000, Brazil; ‡ H. Bremer & Filhos LTDA, Research and Development Department, Rua Lilly Bremer, 322, Rio do Sul, Santa Catarina 89162-454, Brazil

## Abstract

Biomass combustion is critical to Brazil’s energy transition, but high-temperature corrosion remains a major operational barrier in industrial boilers. Following a systematic search of Scopus (2000–2026; 977 records screened, 159 included), this review synthesizes the physicochemical mechanisms driving ash deposition and high-temperature corrosion in biomass-fired systems, identifying chlorine-induced active oxidation and alkali-driven hot corrosion as the dominant degradation pathways, and detailing the synergistic, self-reinforcing interaction between the two. Mitigation strategies:fuel management, additive dosing, protective coatings, and advanced alloys are critically compared on effectiveness, cost, and industrial readiness, showing that no single strategy is simultaneously effective, inexpensive, and mature. Unlike previous reviews, this work integrates a quantitative assessment of the Brazilian industrial landscape, distinguishing a well-documented core (sugar cane bagasse, black liquor) from an undocumented but rapidly growing frontier (rice husk, corn cob), and closes with three priority research gaps: open ash/exposure databases, standardized antislagging and cladding-thickness metrics, and field-validated corrosion prediction, with concrete actions proposed to address each.

## Graphical abstract

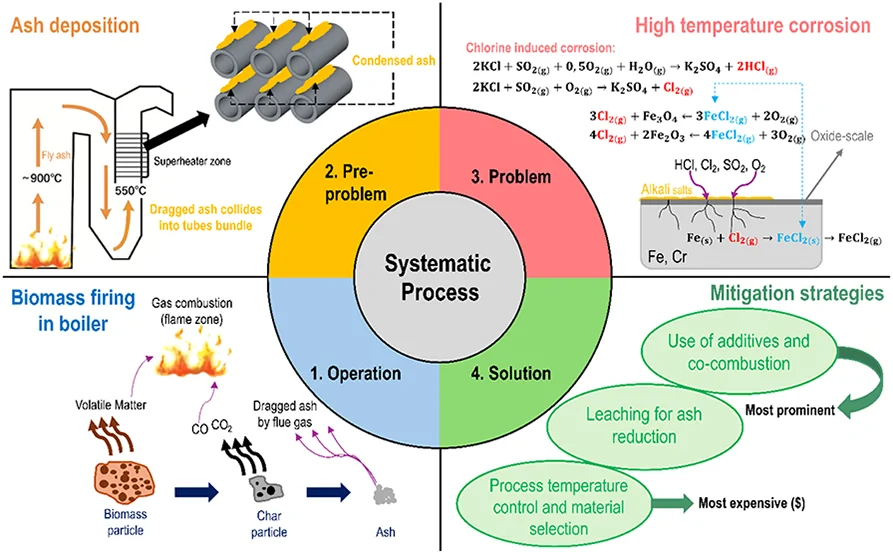

## 1 Introduction

Biomass is a prominent renewable energy source within the context of sustainable urban development. Increasing concerns over greenhouse gas (GHG) emissions, specifically CO_2_, have accelerated the global shift toward renewable energy sources. This transition has been particularly emphasized following the Paris Agreement (COP 21),^1,4^ which established directives aimed at limiting the global temperature increase to 1.5 °C by 2030. Consequently, standalone biomass combustion and its cofiring with coal for electricity and thermal energy generation have been widely investigated in recent years as strategies to mitigate global warming and reduce GHG emissions.^4^ Nevertheless, although CO_2_ emissions experienced a temporary decline during the COVID-19 pandemic due to lockdowns and the ensuing economic downturn,^5^ achieving sustained emission reductions remains a considerable challenge for global economies.^2,3^

Within the renewable energy sector, bioenergy derived from carbon-based materials via direct combustion or thermochemical processes is essential for heat generation. In Brazil, the 2025/26 grain harvest increased by approximately 987.5 thousand tonnes over the previous cycle, reflecting a continuous agricultural expansion that generates vast quantities of biomass residues.^6^ Efficiently processing these volumes is both an economic and sustainable necessity, positioning biorefineries as central to converting such materials into power.^7^ Biomass is broadly defined as organic matter derived from plant and animal activities,^8^ encompassing materials such as rice husks, wood chips, sawdust, vegetable fibers, and sugar cane bagasse, as well as various agricultural and forestry byproducts.^9^

Thermal processing of biomass primarily encompasses pyrolysis and combustion.^10^ Pyrolysis, a thermochemical process that occurs in the absence of oxygen, decomposes organic matter into gaseous, liquid, and solid fractions.^9^ As a fundamental preliminary stage during firing in industrial boilers, this thermal decomposition directly dictates energy efficiency, emission profiles, and the formation of critical byproducts, such as fly ash and corrosive compounds.^11^ Conversely, combustion involves direct oxidation of the material. In boilers, this predominantly occurs on grate systems, a technology specifically designed to efficiently couple primary air distribution with the longitudinal transport of the solid fuel.^12^

Sustainable biomass utilization depends on balancing commercial viability, social benefits, and environmental impact, prioritizing local resource use and greenhouse gas reduction.^13^ Nevertheless, significant challenges remain for industrial steam generators, specifically material degradation driven by ash deposition and accelerated internal corrosion. Biomass combustion generates corrosive flue gases characterized by high alkali and fly ash contents. These species condense and react on cooler internal surfaces, forming low-melting-point compounds that compromise boiler integrity.^14,15^

Corrosion inflicts irreversible damage on metal surfaces, leading to material thinning and imposing substantial economic burdens. Often underestimated in theoretical frameworks, these issues incur annual maintenance costs exceeding US$ 500 million within the steam production sector alone, jeopardizing the economic viability of bioenergy projects.^16^ In Brazil, overall material corrosion costs are estimated at approximately US$ 15 billion annually, representing a significant portion of the national GDP.^17^ In biomass boilers, these financial losses primarily stem from plant downtime and equipment maintenance necessitated by ash deposition and accelerated corrosion.^18^ Specifically, annual maintenance expenditures for solid waste-fired boiler units range from US$ 18,000 to US$ 1.2 million per unit due to corrosion-related failures.^19^ Nevertheless, implementing appropriate mitigation strategies can reduce these annual costs by 25–30%.^20^ Consequently, over the past two decades, numerous studies have explored the application, efficacy, and feasibility of various techniques designed to mitigate corrosion associated with biomass firing in steam generators. However, the absence of updated, boiler-specific cost data for the Brazilian bioenergy sector is identified here as an explicit research gap, addressed further in Section 8.

Despite its recognition as a renewable, carbon-neutral energy source,^21,23^ large-scale biomass utilization is hindered by severe technical challenges, primarily ash deposition and accelerated equipment corrosion that compromise thermal efficiency.^24,25^ This issue is particularly critical in Brazil, where biomass accounts for 32% of the primary energy matrix;^26^ however, technological gaps persist in optimizing the combustion of abundant, heterogeneous agricultural residues such as rice husks and corn cobs. Compared to conventional fuels, the ashes of these materials contain elevated concentrations of potassium (K), chlorine (Cl), and silica (SiO_2_), promoting the formation of aggressive deposits on heat transfer surfaces.^27^

This review is based on a systematic search of the Scopus database (2000–2026) using combined keywords related to biomass combustion, ash deposition, and high-temperature corrosion, following the selection protocol detailed in Section 2. Several reviews have previously addressed high-temperature corrosion in biomass-fired systems (Table 1). Antunes and de Oliveira^28^ provided an early systematic treatment of corrosion mechanisms and material selection but did not address mitigation strategies in comparable depth, nor consider region-specific industrial contexts. Niu et al.^29^ broadened the scope to ash-related phenomena as a whole (slagging, fouling, agglomeration, and corrosion), offering the most comprehensive mechanistic coverage data, yet without a declared search methodology. More recent reviews narrowed the focus to specific submechanisms: Hu et al.^30^ on molten-salt hot corrosion and Li and Zeng^31^ on heat-exchanger materials in co-combustion plants. Moreover, Abioye et al.^15^ and Maj et al.^32^ updated the ash-management and mitigation literature through 2024–2025. None of these reviews, however, combines an explicit, reproducible literature search protocol and a quantitative treatment of the Brazilian industrial landscape, including coverage of emerging mitigation approaches such as FeCrAl alloys, ceramic coatings, and AI-based predictive monitoring. This 3-fold gap is addressed in the present review. Beyond this comparative gap, the corrosion literature for steam generators remains fragmented and, in several aspects, contradictory: studies disagree on the relative aggressiveness of KCl versus K_2_SO_4_ (Section 5.3), on the effectiveness of water-based leaching pretreatments (Section 6.1), and on whether coal cofiring mitigates or worsens biomass-ash corrosivity (Section 6.1). Reconciling these discrepancies, rather than reporting them as isolated findings, is a central objective of the present synthesis.

**Table 1:**
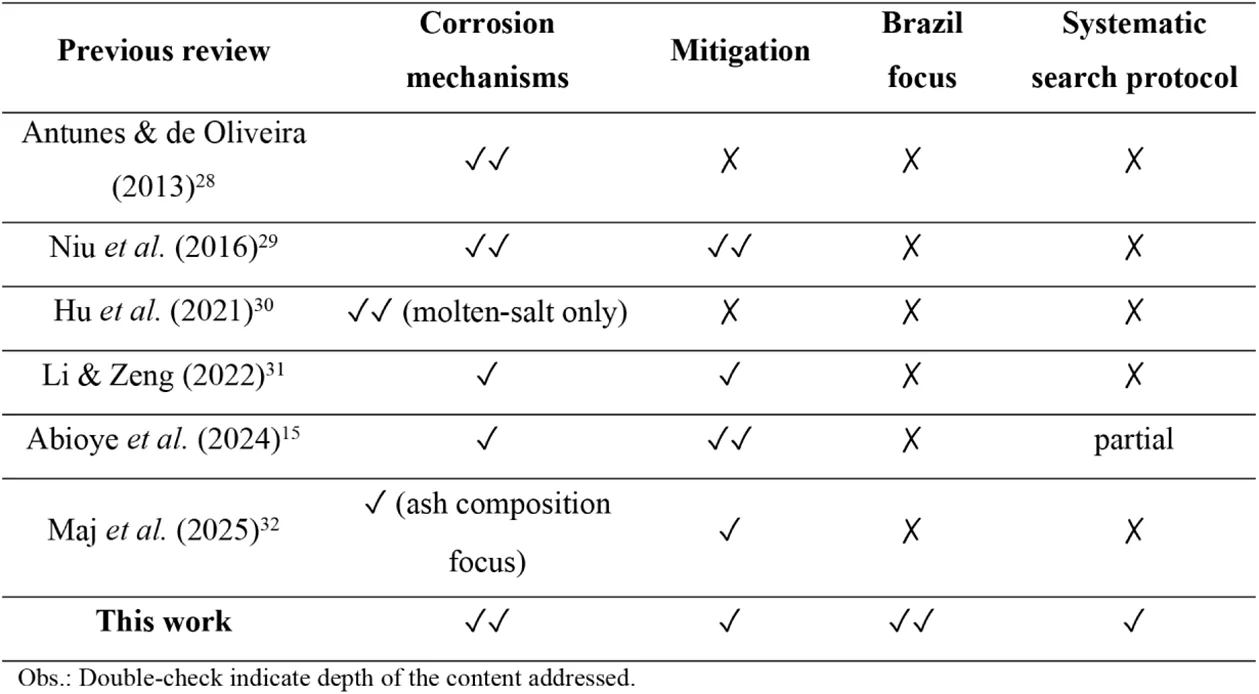
High-Temperature Corrosion Previous Review: Characteristics and Comparison with This Work

A bibliometric analysis of the Scopus database (Section 2) further indicates a sustained increase in publication output on biomass-related high-temperature corrosion since 2011, with an increasing share of studies addressing digital/predictive monitoring and advanced alloys since 2023 (see Section 2.3), underscoring both the rapid evolution of the field and the need for an updated synthesis. Consequently, the primary motivation of this Review is to address the need to understand the operational challenges of biomass combustion in industrial grate boilers, the predominant technology in the manufacturing sector, and to contextualize these issues within the Brazilian sustainable energy landscape. This Review is structured to reflect that range: Section 3 characterizes biomass fuel properties and Brazilian availability; Section 4 details the origin of ash deposition and alkali transformation during thermal processing; Section 5 examines the high-temperature corrosion mechanisms driven by chlorine, molten alkali salts, potassium hydroxides/carbonates, and sulfur; Section 6 critically compares mitigation strategies:fuel management, additive dosing, protective coatings, and advanced alloys on effectiveness, cost, and industrial readiness. Section 7 reviews the experimental methodologies used to evaluate high-temperature corrosion; and section 8 provides a quantitative assessment of the Brazilian industrial landscape. Consequently, the primary motivation of this Review is to address the need to understand the operational challenges of biomass combustion in industrial grate boilers, the predominant technology in the manufacturing sector.

## 2 Review Methodology

To ensure the literature corpus underlying this review, a systematic search was conducted in Scopus using the criteria described in topics 2.2–2.3. The resulting records were cross-checked against the literature already incorporated in this review, and the selection process is summarized in Figure 1 following PRISMA 2020 reporting conventions.^33^

**Figure 1:**
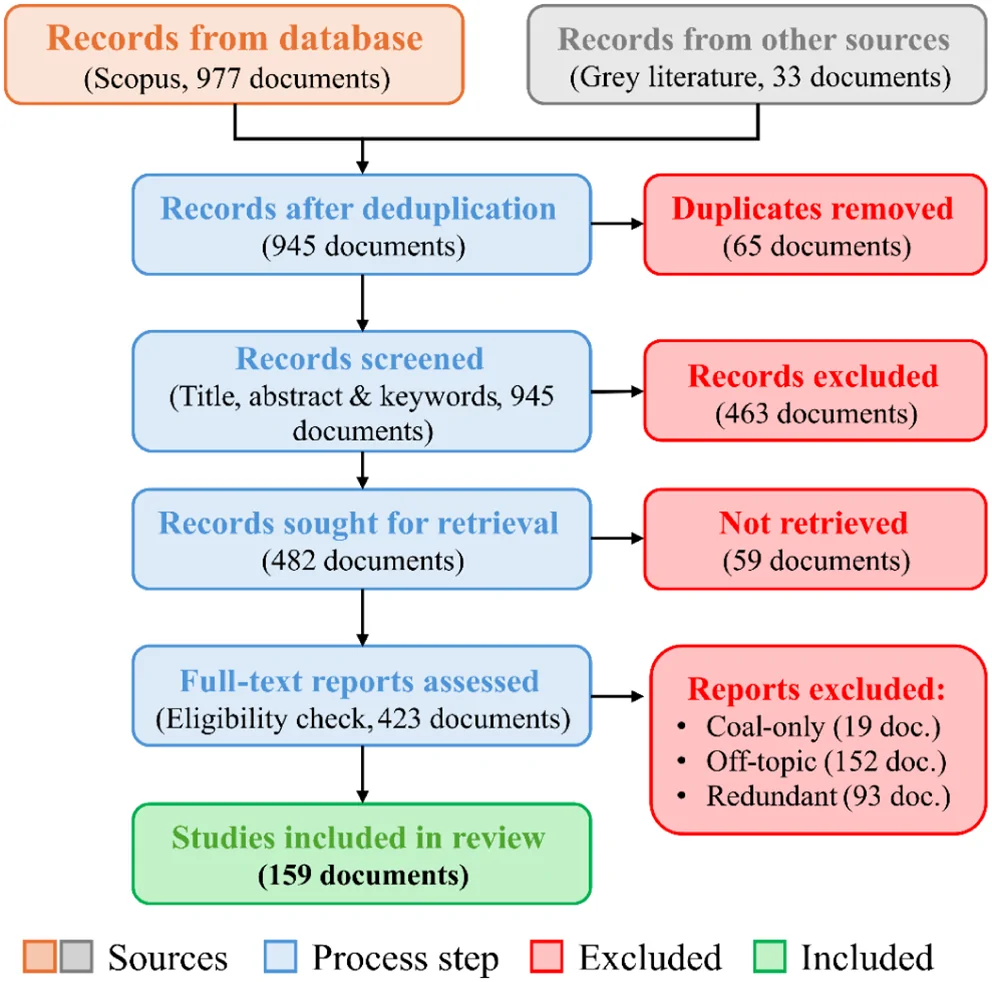
Review methodology based on PRISMA flowchart with specific eligibility criteria.

### 2.1 Information Sources and Search Period

Peer-reviewed literature was retrieved from the Scopus (Elsevier) database, covering publications from 2000 to April 2026. Gray literature relevant to the Brazilian context, such as technical reports from the Brazilian National Agency of Electric Energy (ANEEL), the Energy Research Office (EPE), the Brazilian Corrosion Association (ABRACO), and the Brazilian Industrial Research and Innovation Company (EMBRAPII), was additionally consulted via institutional repositories, given the limited indexing of Portuguese-language technical reports in commercial databases. Scopus was selected as the primary bibliographic database given its broader coverage of Natural Sciences and Engineering journals relative to Web of Science, with the latter’s relevant content largely represented within Scopus’s index. The search string combined terms related to (I) the fuel/process, (II) the degradation phenomenon, and (III) the mitigation approach. Searches were restricted to the Title, Abstract, and Keywords fields, limited to articles, reviews, and conference papers in English and Portuguese.

### 2.2 Eligibility Criteria, Study Selection, and Data Synthesis

Figure 1 illustrates a chart with information source and search period incorporated into the eligibility criteria adopted. The sequential method describes inclusion papers: (I) peer-reviewed studies reporting experimental, field, or modeling data on ash chemistry, deposition, or high-temperature corrosion mechanisms in biomass-fired or cofired systems; (II) studies on mitigation strategies (fuel pretreatment, additives, alloys, coatings, monitoring) applicable to biomass combustion; and (III) studies on the Brazilian biomass energy landscape, regardless of corrosion focus, to support Section 8, and exclusion papers: (I) studies exclusively on coal without a biomass cofiring component; (II) duplicate records across databases; (III) nonpeer-reviewed sources without independent verification (except cited gray literature reports); (IV) records without retrievable full text; and (V) studies whose findings substantially overlapped with mechanisms, data, or conclusions already covered by other included studies, without contributing additional mechanistic insight, quantitative data, or perspective to the synthesis (redundant coverage).

### 2.3 Bibliometric Overview

The search retrieved 977 records. Annual output rose from an average of approximately 6 records/year in 2000–2010, to 34 records/year in 2011–2020, and to 99 records/year in 2021–2025. Furthermore, an approximately 18-fold increase in the mean annual publication rate over 25 years. Publications from the last three complete years (2023–2025) alone account for 338 records, representing 38% of the entire 2000–2025 corpus, while 2024 and 2025 together (259 records) represent 29% of that total. As of the present search, July 2026 already accounts for 87 records despite covering a partial year only, indicating that the field’s growth has not plateaued (see Figure 2).

**Figure 2:**
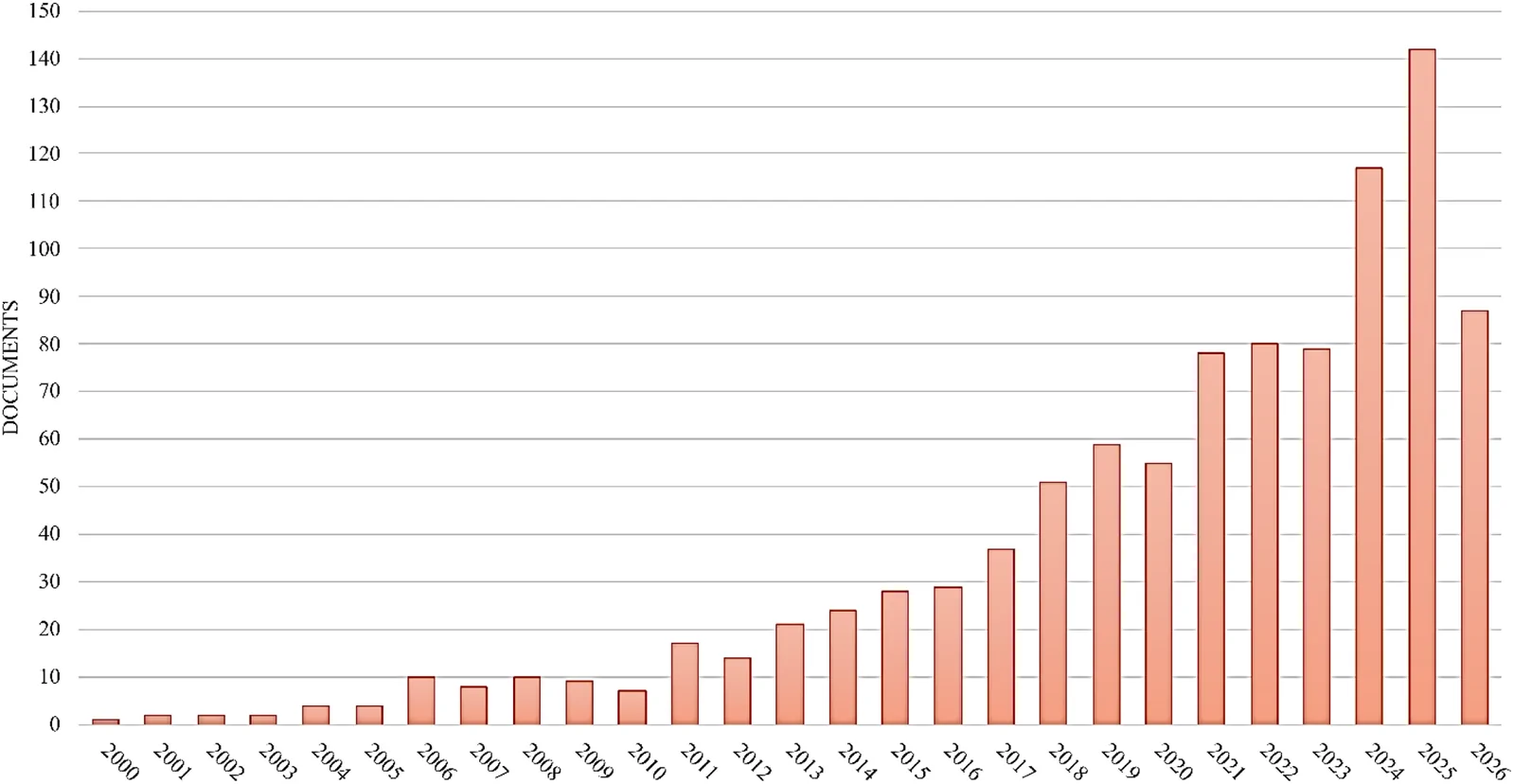
Number of documents by year for alternative fuel processing, degradation phenomenon, and mitigation strategies in the Scopus database.

This pronounced recent acceleration directly supports the need to weigh the present synthesis toward literature, considering that only 8.5% of the bibliometric production is review documents, according to Figure 3a. Furthermore, a distinct concentration of scientific literature is observed in countries like China and India, which closely mirror Brazil in terms of agricultural production and biomass byproduct generation, with this concentration of literature representing 55.3% of the total (see Figure 3b). The scarcity of publications focused on the Brazilian scenario highlights a scientific and technological gap. Left unaddressed, this shortfall could delay national progress and compromise the country’s energy matrix transition, thereby underscoring the necessity of this study.

**Figure 3:**
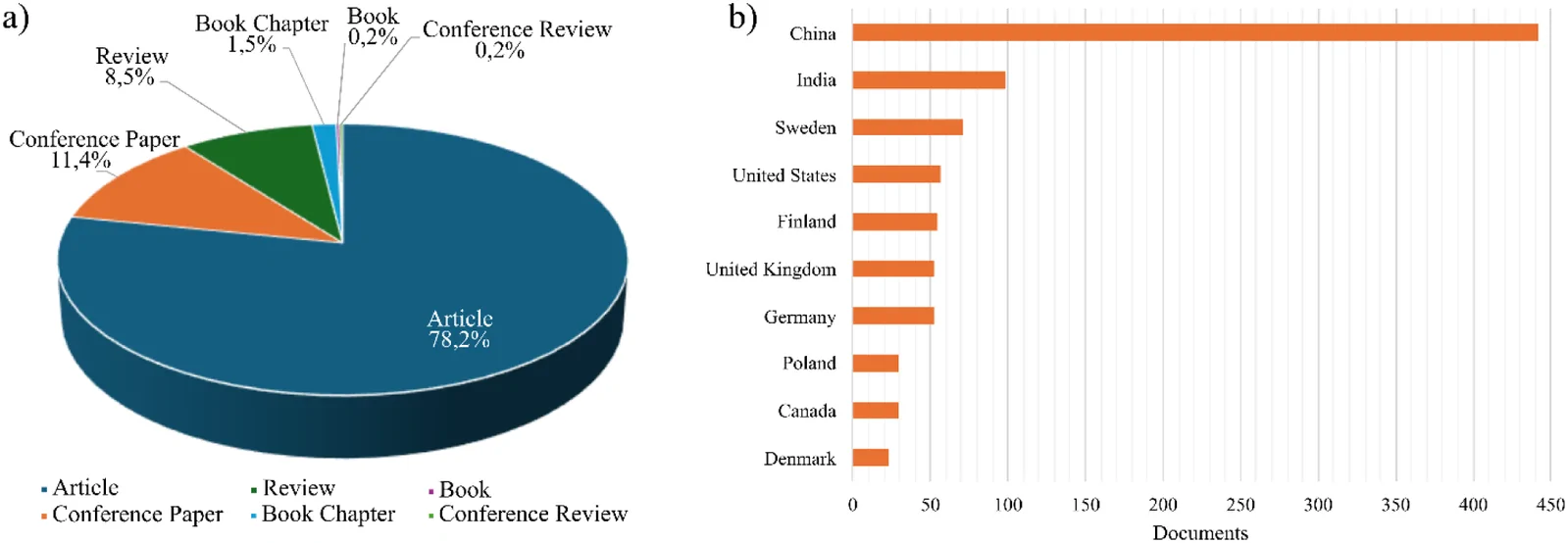
Records analyzed in the Scopus database: a) distribution by types of documents; b) distribution by country or territory.

## 3 Biomass Characteristics and Brazilian Availability

The chemical and structural compositions of biomass vary significantly with its origin, directly affecting its suitability for energy conversion. Agronomic factors, including plant nutritional traits, cultivation methods, soil fertility, and climatic conditions, profoundly influence this composition, a variability clearly reflected in the properties of rice straw and husk.^34–36^ In Brazil, rice is a primary example of this heterogeneity, with approximately 50% of national production concentrated in the state of Rio Grande do Sul.^37^ The vast quantities of husk residue produced in this temperate region exhibit ash properties that differ substantially from those of crops in warmer climates, thereby complicating their standardized evaluation as fuel sources. Agricultural biomass, primarily derived from grain cultivation, generates a substantial quantity of residues. Within the Brazilian context, crop production dynamics vary, with certain commodities experiencing expansion while others demonstrate retraction or stabilization. Table 2 details the primary crops that exhibited significant fluctuations in the latest national census.

**Table 2:** Comparison of Area and Production between the 2024/25 and 2025/26 Harvest Seasons in Brazil^6^

**Crop**	**Area**2024/25 **(thousand ha)**	**Area**2025/26**(thousand ha)**	**Area Change**	**Production**2024/25**(thousand t)**	**Production**2025/26**(thousand t)**	**Production change**
Bean	2,849	2,855	–0.2%	3,268	3,284	–0.5%
Castor bean	69.60	76.10	+9.3%	100.0	147.4	+34.8%
Corn	21,09	22,80	+4.0%	141,0	138,9	–1.5%
Cotton	2,056	2,000	–2.8%	3,853	3,800	–1.4%
Peanut	280.4	281.8	+0.5%	1,079	1,100	–1.9%
Rice	1,758	1,600	–9.9%	12,58	11,10	–13.3%
Sorghum	2,003	1,800	+11.3%	6,100	6,700	+9.0%
Soybean	47,40	48,70	+2.8%	171,3	176,1	+2.7%
Sunflower	61.82	63.80	+3.1%	100.4	101.9	+1.5%

Proximate and ultimate analyses are fundamental for assessing the energy potential and predicting the combustion performance of biomass.^38^ Its primary structural components, cellulose, hemicellulose, and lignin, exhibit distinct thermal decomposition profiles: cellulose degrades primarily between 325 and 410 °C (accounting for approximately 80% of its mass loss), hemicellulose decomposition initiates near 190 °C, and lignin begins to break down above 210 °C.^39^ The depolymerization of these lignocellulosic chains acts as a crucial “activation” pathway for organic matter, driving the subsequent pyrolysis and combustion processes during solid-fuel firing.^40^

However, the thermal conversion of these materials is inherently hindered by severe ash deposition and accelerated corrosion. Consequently, physicochemical evaluations primarily focus on the ash fraction, which concentrates critical inorganic elements such as Na, K, Mg, Ca, Al, and Si in solid and crystalline forms.^35^ Under typical boiler operating conditions, this ash, alongside chlorine and sulfur species, forms aggressive deposits on metallic heat-transfer surfaces.^41,42^ This elemental composition critically dictates the operational performance of conversion systems; for instance, agricultural residues rich in potassium and chlorine are notorious for inducing severe boiler corrosion.^24^ Complementary studies further emphasize that rigorous physicochemical profiling is essential to mitigate ash-related issues and enhance overall energy conversion efficiency.^43,44^ Table 3 details the prevalent inorganic compositions found in the ashes of various biomass feedstocks.^45^

**Table 3:** Chemical Composition of Different Types of Biomass Ash^a^

**Type**	**Biomass**	**Na**_**2**_ **O**	**K**_**2**_ **O**	**MgO**	**CaO**	**Al**_**2**_ **O**_**3**_	**SiO**_**2**_	**ref.**
Woody	Elm bark	0.87	5.47	2.49	83.5	0.12	4.48	^46^
	Olive wood	3.67	25.2	3.03	41.5	2.02	10.2	^47^
	Spruce wood	0.50	9.60	1.10	17.2	9.40	49.3	^48^
	Pine chips	1.20	4.51	2.43	7.89	7.04	68.2	^50^
	Pine pruning	0.42	22.3	11.3	44.1	2.75	7.76	^50^
Range		0.42–3.67	4.51–25.2	1.10–11.3	7.89–83.5	0.12–9.40	4.48–68.2	
Herbaceous/ agriculture	Bamboo	0.31	53.4	6.57	4.46	0.67	9.92	^51^
	Sorghum grass	1.74	9.49	3.12	10.4	0.81	66.9	^52^
	Corn straw	0.16	18.5	4.49	14.7	5.06	50.0	^49^
	Rice husk	0.16	2.29	0.19	0.97	0.21	94.5	^53^
	^a^Rice husk	n.d.	0.72	n.d.	n.d.	0.08	93.5	^54^
	^a^Acai seed	1.70	16.8	-	5.40	7.20	33.8	^55^
	^a^Sugar cane bagasse	n.d.	9.42	4.25	6.51	12.8	45.7	^56^
	Wheat straw	0.60	31.9	1.98	8.08	1.01	41.5	^57^
	Sugar cane straw	0.50	9.50	2.91	6.50	2.05	67.1	^57^
Range		0.16–1.74	0.72–31.9	0.19–6.57	0.97–14.7	0.08–12.8	9.92–94.5	

a Average used in the study was grown in Brazilian territory.

Although Table 3 predominantly features data from foreign cultivars, several feedstocks such as wheat straw, corn stover, rice husks, and sugar cane straw are widely cultivated in Brazil. This disparity highlights a significant literature gap regarding the mineral composition of ashes from Brazilian biomass, reflecting a historical undervaluation of such data for thermal processing. Consequently, knowledge concerning biomass-induced corrosion remains limited within the domestic power sector, severely hindering the deployment of effective prevention and mitigation technologies.^58^

Herbaceous and agricultural feedstocks dominate the Brazilian energy matrix and account for a vast majority of corrosion-related issues. As illustrated in Table 3, these materials exhibit substantially higher average silica and alkali contents compared to woody biomass.

## 4 Origin of the Problem during Biomass Thermal Processing

In Brazil, particularly within the northeastern region, alternative energy strategies have historically centered on the dominance of sugarcane.^52^ Furthermore, at higher energy-demand scales (5 to 10 MW), biorefineries processing substantial volumes of sugarcane for ethanol production are prominent energy contributors.^59^ The diversification of biomass feedstocks has been further explored by Ferreira-Leitão et al.^60^ and Lima et al.,^61^ who highlighted the vast energy potential derived from the immense indirect generation of solid residues by the Brazilian agroindustry. Consequently, direct firing in industrial steam boilers presents a highly viable solution, driven by the abundant availability of these agricultural residues in geographically favored emerging economies such as Brazil.

Regarding the processing of these solid residues, Rocha et al.^62^ and de Luna et al.^63^ explored the energy potential of biomass through thermogravimetric analysis (TGA), identifying high volatile and organic matter contents that support its suitability as a fuel. These biofuels can be produced via pyrolysis and further upgraded through torrefaction, a process that enhances energy density by increasing the carbon content.^64^ Nevertheless, energy performance is not determined by carbon content alone; it is also significantly hindered by the ash content inherent to lignocellulosic fuels.^65,66^

Thermochemical processes generate substantial amounts of heat, steam, or electricity, while enabling the production of combustible bioproducts such as syngas, bio-oil, and biochar.^67–70^ Furthermore, boilers are conventional systems for steam generation, designed either for direct fuel firing or for yielding value-added products through thermochemical routes like pyrolysis and gasification.^71–73^ As feedstocks undergo stages of pyrolysis, gasification, and combustion, the characteristics of the resulting reaction products are fundamentally modified. This transformation can significantly compromise equipment integrity, particularly in zones susceptible to fly ash accumulation, such as superheaters and economizers.

Direct firing in boilers remains the most widely adopted industrial process because of its operational simplicity. The fuel is oxidized to generate high-temperature flue gases that heat the water and produce steam. Similar to combustion, gasification systems necessitate regular maintenance due to the formation of tars (undesirable heavy hydrocarbons) that can precipitate and induce severe fouling on heat-transfer surfaces. During gasification, biomass is converted into a combustible syngas suitable for boiler applications; however, this gas inherently retains corrosive compounds that can detrimentally impact the fuel conversion process itself.^74^

Compared to alternative configurations, grate-firing technology exhibits lower conversion efficiency. This is primarily attributed to the higher excess-air requirement—typically 5–8% O_2_ in the flue gas—necessary to ensure complete combustion.^75^ For instance, while 20 MWe fluidized-bed units equipped with modern steam cycles can achieve electrical efficiencies of 30–35%, comparably sized grate-fired boilers generally operate at lower efficiency thresholds.^76^

However, grate-boiler operation faces substantial challenges predominantly linked to biomass ash chemistry, specifically slagging, fouling, and corrosion of internal components.^15^ Research in this area remains sparse in Brazil. Recently, Brand et al.^77^ identified high fouling potential in ash samples from the drag conveyor and settler of a 28 MWe industrial boiler in Southern Brazil, while also highlighting the risk of alkali-induced corrosion. Their findings suggest that grate-firing operational conditions intensify ash-deposition issues. Nevertheless, the study did not employ advanced methods to qualify or quantify corrosion mechanisms, revealing a critical knowledge gap within the national context.

Furthermore, ash-related issues in biomass combustion represent critical technoeconomic challenges rather than mere operational inconveniences. These phenomena dictate fuel selection, plant design, and material choice, ultimately determining the economic viability of power generation. Ash deposition reduces heat transfer rates and thermal efficiency while triggering premature, unscheduled plant shutdowns for maintenance due to material damage.^78^ The primary degradation mechanism involves alkali chloride chemistry, specifically potassium chloride (KCl). Released significantly during agricultural biomass firing, KCl acts as an initiator for adhesive deposit formation and a catalyst for accelerated high-temperature corrosion, severely limiting the service life of critical components such as superheaters.^29,79^

### 4.1 Ash and Alkali Transformation Pathways

The pathway of inorganic elements from the solid fuel to the formation of deposits or emissions is a complex and highly debated process governed by the fuel composition, the combustion technology, and, above all, the local temperature. This section first describes the physical fractionation routes by which alkali species partition among bottom ash, fly ash, and the gas phase (Section 4.1.1) and then details how the dominant potassium species and their transformation reactions evolve across successive temperature regimes (Section 4.1.2).

#### 4.1.1 Volatilization and Chemical Fractionation Routes

Ash generation is generally categorized into distinct mechanisms: volatilization, bottom ash formation, and fly ash entrainment. At typical grate-firing temperatures (1000–1200 °C), a substantial fraction of alkali metals, along with virtually all chlorine (Cl) and sulfur (S), are released into the gas phase.^80^ This behavior highlights a crucial distinction from conventional coal combustion, where inorganic constituents are predominantly retained within a glassy ash matrix. The primary volatilized species formed are alkali chlorides and hydroxides (NaCl, KCl, and KOH).^81^

In grate-fired systems, the design intent is for the bulk of the ash to be discharged as bottom ash.^82^ Nevertheless, a fraction is inevitably entrained by the flue-gas flow as coarse fly ash, while the volatilized species nucleate and condense into submicrometer aerosols upon cooling.^83^ Consequently, severe chemical fractionation occurs: the bottom ash becomes heavily enriched in calcium (Ca) and silica (Si), whereas the fly ash and subsequent deposits are disproportionately rich in problematic volatile elements (K, Cl, and S).^84^ This chemical partitioning is a fundamental driver of accelerated degradation observed in biomass boilers.

Alkali metals are widely recognized in the literature as the primary agents of boiler corrosion, particularly affecting superheaters at high temperatures. The presence of these elements in biomass feedstocks is a decisive factor driving the oxidation of metallic tubes.^28^ During combustion, alkali-rich particles are generated and subsequently deposit on superheater surfaces.^15^ Early research in the 1980s elucidated the initial oxidation pathways induced by alkali metals on boiler tubes during waste incineration.^85^ Following these foundational studies, by the late 1990s, the focus regarding alkaline species shifted to agricultural biomass firing, prompting experimental investigations into the combined effects of potassium and chlorine concentrations within corrosive aerosols.^86^

#### 4.1.2 Temperature-Dependent Alkali Transformation Regimes

The dominant chemical form of alkali species, and therefore their corrosive potential, is not fixed but evolves continuously with temperature. Consolidating the mechanisms reported across recent studies, the transformation of potassium, the most abundant and problematic alkali in agricultural biomass, can be divided into three successive thermal regimes, each governed by distinct reactions:

(1) Low-temperature regime (300–600 °C). During the initial pyrolysis stage, potassium initially present as water-soluble KCl reacts with the active functional groups (carboxylic and phenolic) generated from the decomposing organic matrix. Chen et al.^87^ demonstrated that this reaction converts a fraction of the mobile KCl into water-insoluble, organically bound potassium, releasing gaseous HCl in the process. The extent of this conversion is governed jointly by the availability of active functional groups produced during pyrolysis and by the amount of KCl present; at 400 °C, the water-insoluble fraction reached a maximum, accounting for an estimated of the total potassium in samples of increasing initial K content. This regime therefore acts as a temporary retention mechanism, transiently binding potassium within the char and delaying its release to the gas phase.
(2) Intermediate-temperature regime (600–900 °C). As the temperature rises above 600 °C, the organically bound potassium becomes unstable and decomposes to form potassium carbonate (K_2_CO_3_).^87^ This species is particularly aggressive, as it readily forms low-melting eutectic compounds on metallic surfaces and, upon reaction with water vapor, generates gaseous KOH, both of which are potent corrosive agents (Section 5.3). The presence of an oxidizing atmosphere was found to favor K_2_CO_3_ generation, whereas the carbonate itself begins to decompose above 800 °C, progressively releasing potassium as K atoms or KOH into the gas phase. Concurrently, in this range virtually all chlorine and a large fraction of the sulfur are volatilized, establishing the gas-phase inventory of KCl, KOH, and sulfur oxides that subsequently drives deposit formation on cooler surfaces.
(3) High-temperature regime (>900 °C). At the temperatures characteristic of grate-firing combustion zones, the release of potassium to the gas phase becomes nearly complete. In a complementary investigation, Mörtenkötter et al.^88^ quantified this behavior across ten biofuels and multiple combustion technologies, showing that almost no potassium is released at fluidized-bed temperatures (below ∼900 °C), whereas the bulk of the release occurs precisely within the 1000–1400 °C window typical of grate systems, becoming complete at roughly 1500 °C. Their work identified grate-firing as the technology most susceptible to severe alkali volatilization, and consequently the primary target for the mitigation strategies discussed in Section 6. Within the gas phase, the partitioning between KCl and K_2_SO_4_ is temperature-sensitive: Wang et al.^83^ observed that potassium sulfation is favored at intermediate temperatures (around 1000 °C), with sulfur being progressively released from the condensed phase above 1100 °C and completely above 1200 °C, so that KCl becomes the dominant volatile potassium carrier at the highest temperatures. Both studies corroborate that temperature remains the dominant factor governing potassium release, dictating the specific reaction mechanisms within each thermal range.

Accordingly, Figure 4 summarizes these transformation routes schematically with a schematic diagram of ash transformation initiated by fuel fragmentation during pyrolysis and combustion. It illustrates the volatilization of soluble alkali, sulfur, and chlorine into the gas phase, followed by their nucleation and condensation into entrained fine particles. Furthermore, the diagram depicts how the introduction of aluminosilicate-based additives captures these nucleated alkaline species, forcing their precipitation into high-melting-point bottom ash.

**Figure 4:**
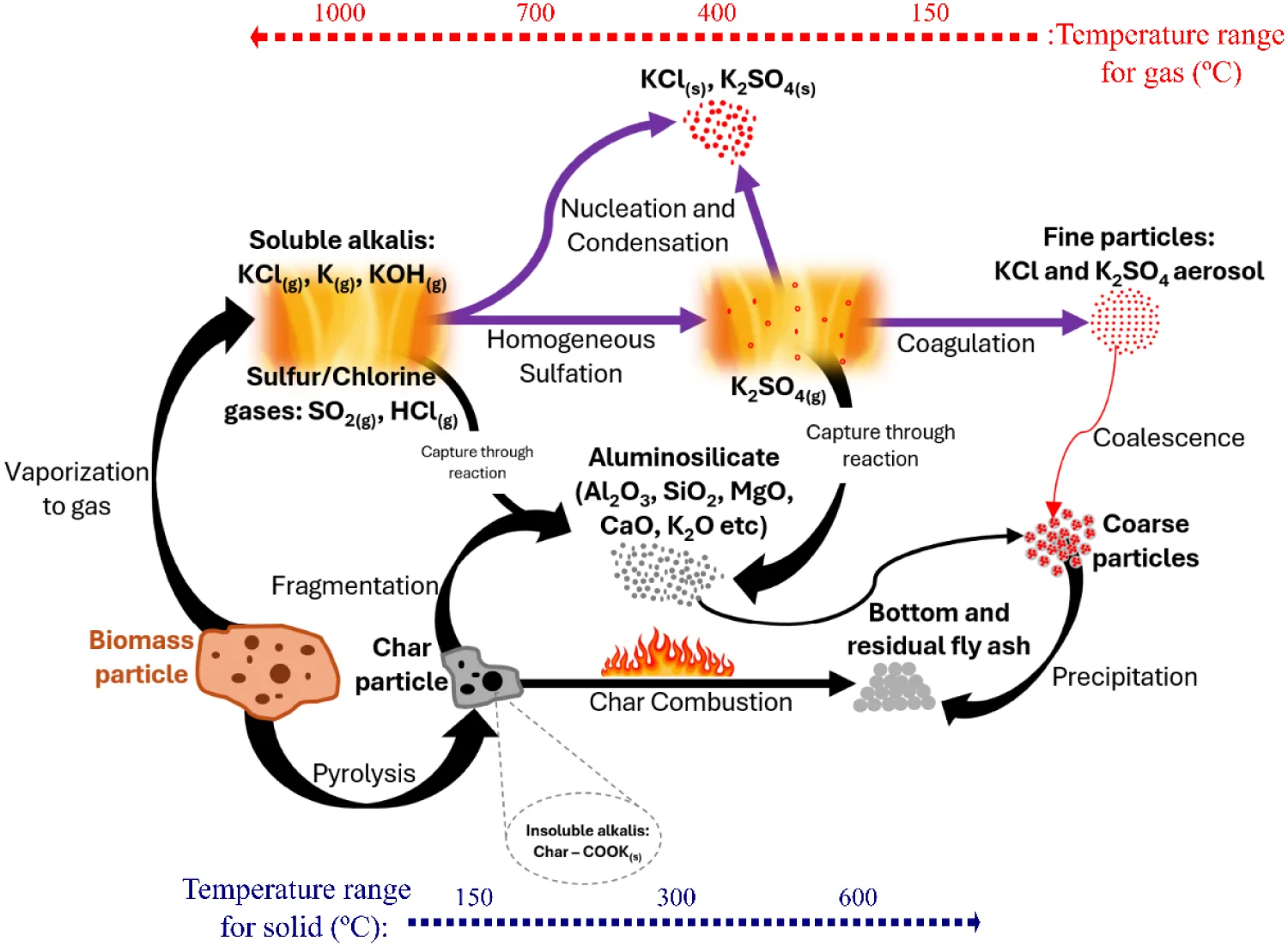
Biomass particle transformation during combustion in boilers. Adapted from Journal of the Energy Institute, Vol. 91, X. Wang, Z. Hu, A. Adeosun, B. Liu, R. Ruan, S. Li, H. Tan, Particulate matter emission and K/S/Cl transformation during biomass combustion in an entrained flow reactor, Pages 835–844, Copyright (2017), with permission from the Energy Institute.

This temperature-resolved framework explains why superheater corrosion is governed less by the peak furnace temperature than by the specific thermal window to which each surface is exposed, and provides the mechanistic basis for the deposition and corrosion pathways detailed in the following sections.

### 4.2 Multiphase Deposition Process on Convective Surfaces

The physicochemical properties of biomass ash favor the formation of deposits within the boiler internals. The low porosity of these particles confers smooth surface characteristics and viscous behavior, resulting in high adhesion capacity and making them difficult to remove.^89^ Ash deposition on tubes results from a combination of three transport mechanisms: (1) inertial impaction dominates mass gain, with turbulent effects promoting accumulation through vortex formation; (2) subsequent heterogeneous condensation reduces the surface gas concentration and facilitates thermophoretic transport between layers; and (3) the process is sustained by the continuous diffusion of inorganic vapors.^90^

Larger particles (>10 μm) with sufficient momentum fail to follow the gas streamlines around the tube and directly impact its surface. Conversely, smaller particles (<1 μm) subjected to a thermal gradient, are driven from the hot flue gas toward the cooler tube surface via thermophoresis. These observed phenomena precede the chemical attack on the metallic surface and can be effectively investigated using computational fluid dynamics (CFD) techniques.^91^ As the flue gas containing volatilized species (e.g., KCl, K_2_SO_4_, and KOH) cools upon contacting cold surfaces such as superheaters, economizers, and air preheaters, these species tend to become supersaturated and condense. This process, in turn, promotes the formation of a thin, sticky, salt-rich initial layer. The condensation of Na/K double sulfates, such as K_3_Na­(SO_4_)_2_, has been identified as a key initiating step in this process.^92^ Over time, the deposit layer thickens and matures; consequently, the continuous heat flux causes the salts within the deposit to melt. This creates a liquid phase that promotes sintering, rendering the deposit increasingly difficult to remove.^84^ The low melting point of alkali chlorides (approximately 770 °C for KCl) and eutectic salt mixtures are highly critical in this context.^93^ Figure 5 presents a schematic representation of the ash deposition mechanism on tube surfaces, according to the mechanisms described.

**Figure 5:**
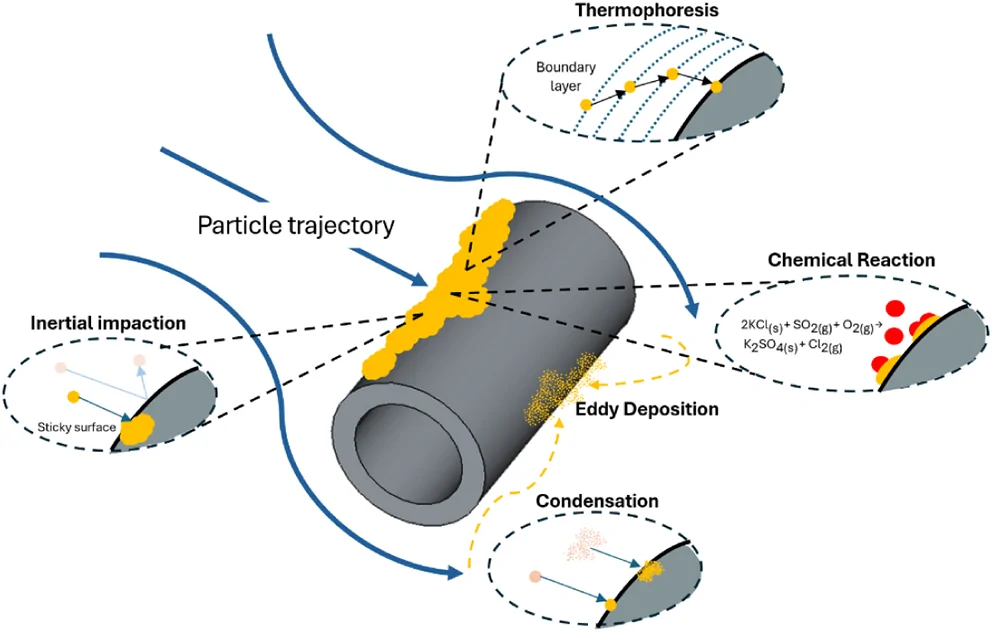
Adhesion of volatile ash and the process of forming developed layers of deposits. Adapted from ref [90]. Copyright 2018 The Authors. Published by Elsevier Ltd. under a CC BY license.

This phenomenon is frequently associated with deposits on convective surfaces at high temperatures, such as superheaters, and serves as a precursor to corrosion induced by chlorine and molten salts. However, corrosion issues in biomass combustion also extend to lower-temperature conditions, commonly referred to as cold-end or low-temperature corrosion, affecting other boiler regions. While both problems stem from ash behavior, their underlying mechanisms differ significantly. Figure 6 provides a schematic differentiating the regions affected by these two phenomena. High-temperature corrosion, the primary focus of this Review, results from degradation driven by the reactivity of alkali species and the presence of highly oxidizing gases. Conversely, cold-end corrosion mechanisms involve acid condensation and the formation of deliquescent salts, which operate primarily through electrochemical processes influenced by flue gas humidity.^94–97^

**Figure 6:**
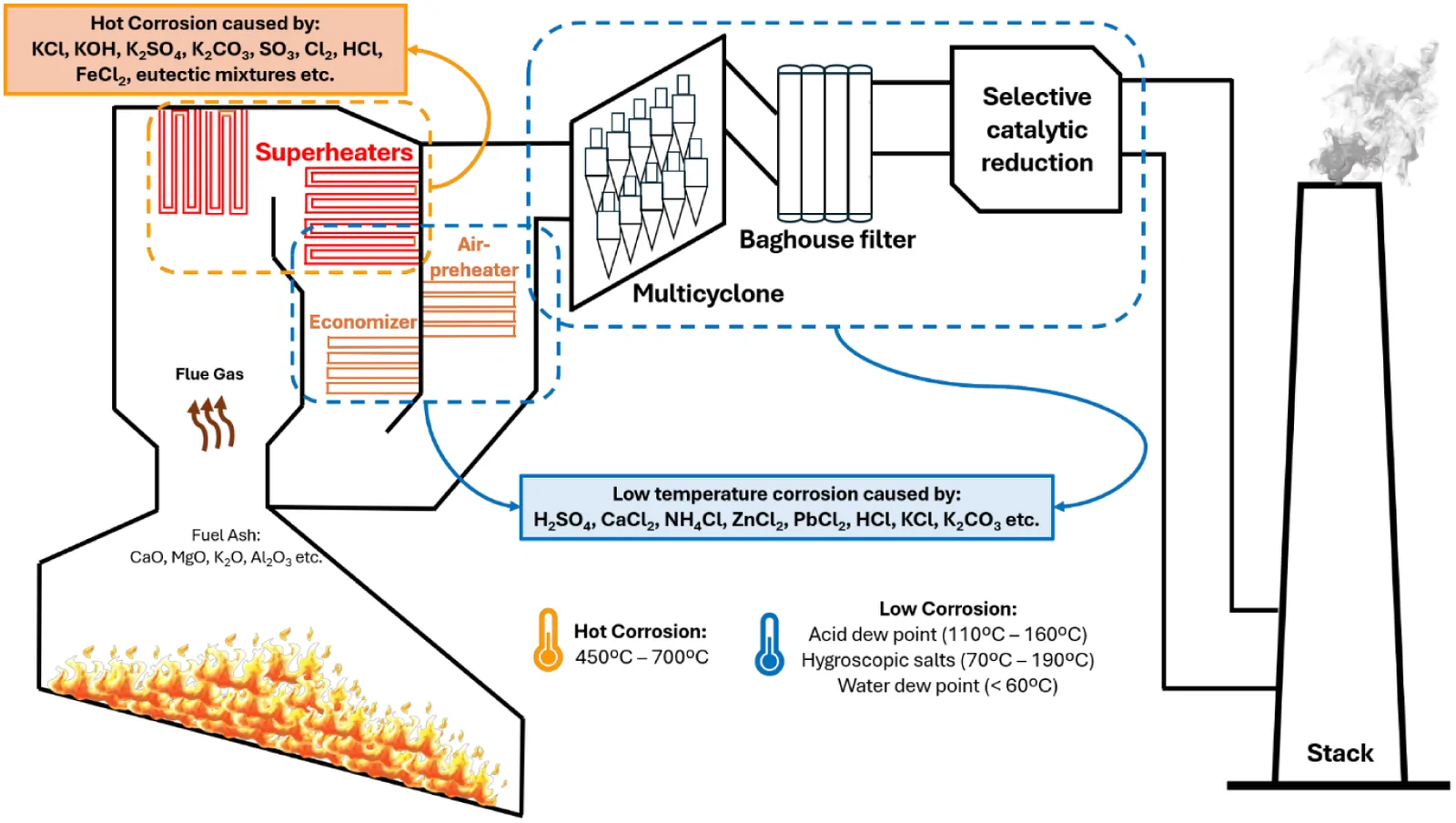
Corrosion regions caused by high- and low-temperature phenomena in steam generators.

## 5 High-Temperature Corrosion: Alkali and Chlorine Pathways

High-temperature corrosion represents the most prominent and economically significant degradation challenges in steam generators firing biomass rich in aggressive elements. It occurs predominantly in superheaters and reheaters, where metallic surfaces are exposed to a complex thermochemical environment.^98,99^ Decoding the underlying mechanisms necessitates a thorough analysis of ash constituents, the associated chemical reactions, and the experimental methodologies employed to simulate these conditions.^100^

### 5.1 Chlorine-Induced Active Oxidation Mechanism

The aggressiveness of a boiler environment is dictated by the inorganic composition of fuel. Various elements, acting individually or synergistically, drive accelerated degradation of materials. Chlorine is universally recognized as the primary catalyst for high-temperature corrosion in biomass boilers.^101^ During combustion, it is released from the fuel in multiple stages, including low-temperature desorption, the cleavage of organic bonds, and the decomposition of inorganic compounds at high temperatures.^102^ Even low chlorine concentrations (<0.1 wt %) in biomass feedstocks can initiate accelerated corrosion, while higher amounts (<0.3 wt %) pose significant environmental risks and toxic emission concerns, specifically regarding polychlorinated dibenzo-p-dioxins (PCDD) and polychlorinated dibenzofurans (PCDF).^103^

Björkman and Strömberg^104^ investigated chlorine emissions during pyrolysis and gasification, demonstrating that chlorine release does not follow a simple physical model based on pure salt volatility. Instead, they showed that chemical interactions with the biomass matrix itself are the decisive factors for low-temperature release. While their work established the “what” (the occurrence of low-temperature release), it left the “why” as an open question. A significant advancement came from Johansen et al.,^105^ who detailed a two-stage mechanism for chlorine and sulfur release and established a strong correlation between potassium release and chlorine availability. In this framework, chlorine was defined as the primary “carrier agent” for potassium into the gas phase, whereas silicates act as the main “retention agent” for the potassium remaining in the ash. More recent research has shifted toward quantitative prediction; for instance, Cao et al.^25^ proposed a model to quantify the formation of specific potassium species (KCl, KOH, and K_2_SO_4_) based on the initial biomass composition. Their work highlights competitive interactions, showing how the presence of chlorine inhibits the formation of KOH and K_2_SO_4_, and how sulfur promotes potassium sulfation at high temperatures.

Active oxidation is the most critical degradation factor limiting the service life and efficiency of biomass-fired steam generators. The presence of chlorine drastically accelerates corrosion rates at metal temperatures exceeding 450–500 °C^32^ This mechanism involves a catalytic cycle that destroys the protective oxide scale on steels.^106^ The foundational reactions for this process, as detailed in the seminal work of Grabke et al.^107^ and further discussed in the literature,^28^ proceed according to the stages described below. eqs 1 and 2 represent the direct attack of gaseous hydrogen chloride (HCl) and chlorine gas (Cl_2_) on metallic iron. eq 3 describes the volatilization of ferrous chloride (FeCl_2_), which exhibits high vapor pressure at typical operating temperatures (∼500 °C). Consequently, this compound diffuses through cracks, pores, and microvoids in the oxide structure and, in the presence of oxygen, reacts to form iron oxides, as shown in eqs 4 through 6.

1 $${\mathrm{Fe}}_{\left(s\right)}{+\mathrm{2HCl}}_{\left(g\right)}\to {\mathrm{FeCl}}_{2(s)}{+H}_{2(g)}$$

2 $${\mathrm{Fe}}_{\left(s\right)}{+\mathrm{Cl}}_{2(g)}\to {\mathrm{FeCl}}_{2(s)}$$

3 $${\mathrm{FeCl}}_{2(s)}↔{\mathrm{FeCl}}_{2(g)}$$

4 $${\mathrm{4FeCl}}_{2(g)}{+3O}_{2(g)}\to {\mathrm{2Fe}}_{2}O_{3(s)}{+\mathrm{4Cl}}_{2(g)}$$

5 $${\mathrm{3FeCl}}_{2(g)}{+2O}_{2(g)}\to {\mathrm{Fe}}_{3}O_{4(s)}{+\mathrm{3Cl}}_{2(g)}$$

6 $${\mathrm{FeCl}}_{2(g)}{+O}_{2(g)}{+\mathrm{Fe}}_{3}O_{4(s)}\to {\mathrm{2Fe}}_{2}O_{3(s)}{+\mathrm{Cl}}_{2(g)}$$

These transformations directly lead to the production of Cl_2_, which exhibits high activity at elevated temperatures due to enhanced gaseous diffusion. Furthermore, Wang et al.^22^ highlighted the critical role of ferrous chloride (III) (FeCl_3_) production; given its low melting point (303 °C) and high vapor pressure (1670 Pa), this compound volatilizes rapidly, creating voids in the protective oxide scale and further intensifying corrosive activity, as described by eq 7–9. Under specific conditions, Cl_2_ exists in equilibrium with HCl and can be converted via the Deacon reaction (eq 10). This facilitates a phenomenon known as chlorine-induced propagation, which reinitiates the entire active oxidation process.^108,109^

7 $${\mathrm{2Fe}}_{\left(s\right)}{+\mathrm{3Cl}}_{2(g)}\to {\mathrm{2FeCl}}_{3(g)}$$

8 $${\mathrm{2FeCl}}_{2(g)}{+\mathrm{Cl}}_{2(g)}\to {\mathrm{2FeCl}}_{3(g)}$$

9 $${\mathrm{4FeCl}}_{3(g)}{+3O}_{2(g)}\to {\mathrm{2Fe}}_{2}O_{3(s)}{+\mathrm{6Cl}}_{2(g)}$$

10 $${\mathrm{4HCl}}_{\left(g\right)}{+O}_{2(g)}⇌{\mathrm{2Cl}}_{2(g)}{+2H}_{2}O_{\left(g\right)}$$

Temperature directly governs active oxidation; thus, its precise control is fundamental to mitigating this phenomenon. Okoro et al.^99^ investigated this issue at a laboratory scale, evaluating the progression of surface reactions on specimens doped with significant KCl concentrations (0.75 mg/mm^2^). Their findings demonstrated that the thickness of the corrosion product layer increases with temperature. Furthermore, thermal fluctuations, such as unexpected cooling events, can be even more detrimental, as the elevated partial pressure of chlorine at high temperatures sustains the corrosive attack for prolonged periods. Conversely, Santos^110^ elucidated that if the metallic surface temperature exceeds 700 °C, the condensation of KCl and NaCl is thermodynamically inhibited. The salts remain in the gas phase and are entrained by the flue gas, thereby preventing the formation of the initial deposit requisite for the attack. Consequently, the assumption that “hotter is always worse” is misleading; the molten salt corrosion rate exhibits a “bell-shaped curve” relative to the metal surface temperature, peaking within an intermediate range, typically between 500 and 700 °C. However, from an energy recovery perspective, operating at excessively high temperatures compromises the fundamental purpose of heat exchangers, particularly superheaters, rendering the simplistic approach of merely increasing local temperatures impractical. Concurrent with the active oxidation cycle, KCl can react directly with protective oxides formed by alloying elements, most notably chromium (Cr), leading to the dissolution of these oxides and the subsequent formation of chromates, as described by eq 11 and 12.^111^ Figure 7 illustrates the process of chlorine-induced active oxidation and chromate formation in superheating metal tubes.

11 $${\mathrm{8KCl}}_{\left(s\right)}{+\mathrm{2Cr}}_{2}O_{3(s)}{+5O}_{2(g)}⇌{4K}_{2}{\mathrm{CrO}}_{4(s)}{+\mathrm{4Cl}}_{2(g)}$$

12 $${\mathrm{4KCl}}_{\left(s\right)}{+\mathrm{2Cr}}_{2}O_{3(s)}{+4O}_{2(g)}\to {2K}_{2}{\mathrm{Cr}}_{2}O_{7(l)}{+\mathrm{2Cl}}_{2(g)}$$

**Figure 7:**
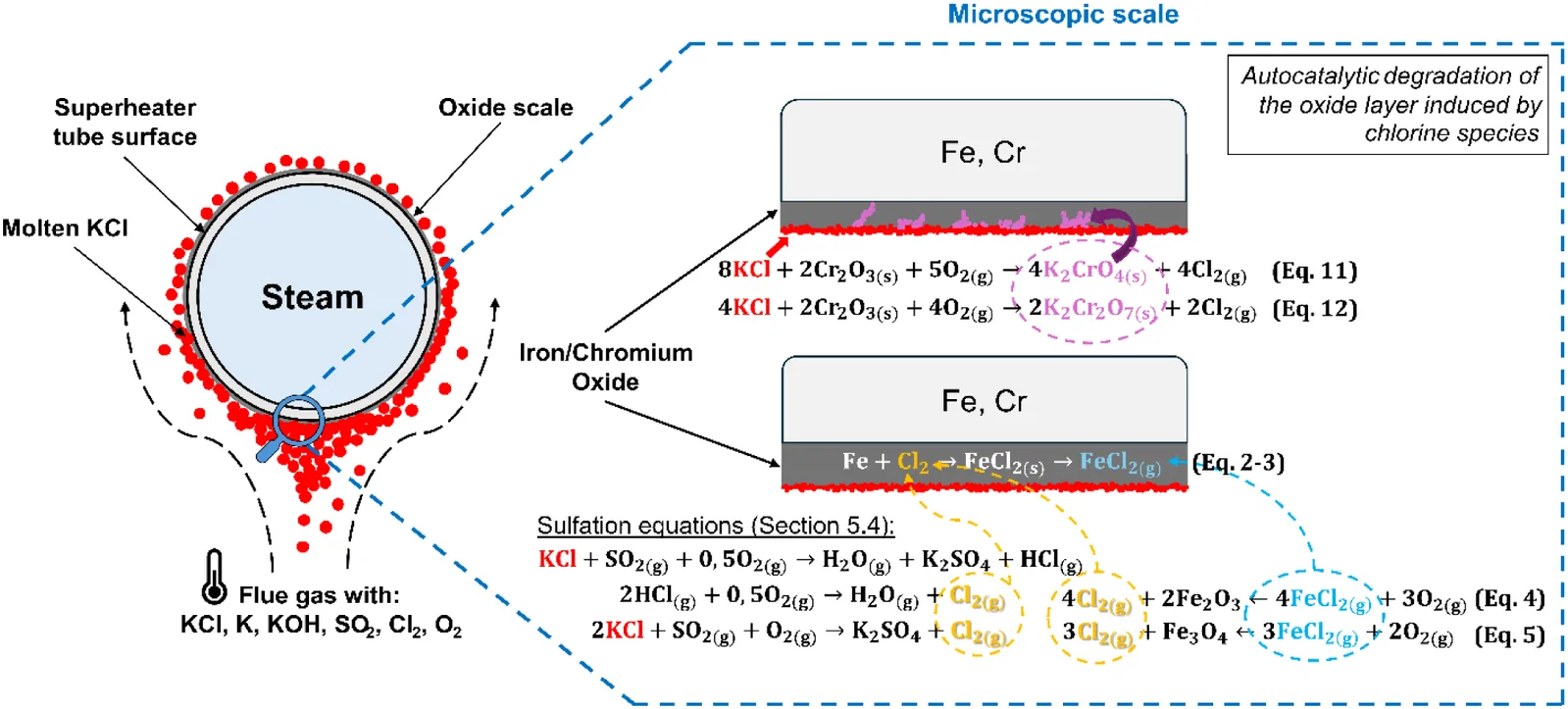
Schematic representation of the chlorine-induced active oxidation mechanism in superheater tubes. Molten KCl deposited on the oxide scale sustains an autocatalytic cycle in which chlorine attacks the metal to form volatile iron chlorides that reoxidize to release Cl_2_. Adapted from Renewable and Sustainable Energy Reviews, Vol. 161, K. Li, Y. Zeng, Corrosion of heat exchanger materials in cocombustion thermal power plants, 112328, Copyright (2022), with permission from Elsevier.

The product of eq 12 is especially hazardous due to its capacity to form a low-melting eutectic mixture with KCl at 368 °C. Both reactions are doubly destructive; they actively consume the protective scale, compromising its structural integrity and leaving the underlying metal vulnerable to subsequent attack.^112^ Consequently, chlorine- and alkali-induced corrosion mechanisms are not independent events but rather synergistic and self-reinforcing processes. While the alkali attack breaches the protective oxide “shield,” the chlorine-active oxidation cycle exploits these vulnerabilities to catalytically attack the metal substrate. This synergy of “shield breaching and direct attack” explains the exceptionally high degradation rates typically observed in biomass-fired superheaters.

### 5.2 Oxidation Mechanism via Molten Salt Formation

Corrosion severity increases drastically once the metal surface temperature exceeds the melting point of the ash deposits. This molten salt layer acts as both an electrolyte and a fluxing agent, rapidly dissolving the protective oxide scale. This mechanism, known as molten salt corrosion or hot corrosion, is significantly faster and more destructive than gas-phase corrosion, leading to unacceptably high material loss rates even for high-performance alloys.^112^ A significant gap in the literature regarding the fundamental aspects of molten sulfate-induced corrosion and the development of effective anticorrosion coatings was addressed in the review by Hu et al.^30^ The authors established that the formation of sulfate salts (primarily Na_2_SO_4_) results from the oxidation of sulfur- and alkali-containing impurities present in the fuels. A simplified mechanism for the formation of these salts, proposed by the authors based on the studies of Stringer^113^ and Eliaz et al.,^114^ can be described by eq 13. This reaction controls the melt chemistry, and the thermodynamic equilibrium conditions of the species follow eq 14.

13 $${\mathrm{Na}}_{2}{\mathrm{SO}}_{4(s)}⇌{\mathrm{Na}}_{2}O_{\left(s\right)}{+\mathrm{SO}}_{3(g)}$$

14 $$\log\left(\alpha_{{\mathrm{Na}}_{2}O}\right)+\log\left(P_{{\mathrm{SO}}_{3}}\right)=\mathrm{‐16},7=\frac{{\Delta G}_{22}^{o}}{2,303.\mathrm{RT}}$$

The parameter -log­(*α*Na_2_O) defines the basicity/acidity of the melt: values exceeding 8.4 correspond to a basic melt, while those below 8.4 indicate an acidic melt. The term $\log\left(P_{{\mathrm{SO}}_{3}}\right)$ denotes the concentration of sulfur trioxide (SO_3_) in the gas phase in terms of partial pressure, and ΔG°_22_ represents the Gibbs free energy associated with the thermodynamic equilibrium of the reaction.

Sulfur-rich environments exert both direct and indirect effects on corrosion mechanisms, influencing both active oxidation and molten salt processes. In this context, Lehmusto et al.^115^ explored a specific corrosion mechanism in heat recovery boilers from copper and nickel smelting. The authors demonstrated that process dust is not inert but acts as a potent catalyst for SO_3_ formation. Consequently, SO_3_ concentrations significantly exceeding thermodynamic equilibrium predictions are the primary cause of sulfuric acid corrosion in these systems. Furthermore, Liet al.^116^ expanded the understanding of the role of sulfates and their relationship with chlorine gas release. They found that minor fluctuations in HCl and sulfur dioxide (SO_2_) concentrations, along with the presence or absence of H_2_O, are the most critical factors affecting corrosion, thereby elucidating the “coupling effect” between these mechanisms. Overall, these studies indicate that oxidation mechanisms can occur simultaneously and through complementary pathways.

The formation of specific critical compounds that destroy protective layers is a key focus in studies addressing biomass combustion, particularly regarding the corrosive role of potassium compounds. The formation of potassium pyrosulfate (K_2_S_2_O_7_), KCl, K_2_SO_4_, and iron–potassium trisulfates (K_3_Fe­(SO_4_)_3_) accelerates the dissolution of protective oxide scales, thereby intensifying the corrosive process.^31^ Furthermore, the occurrence of eutectic salts and chromate compounds is highly relevant when using metallic alloys with varying Cr contents.^111^,eq 15 and[Disp-formula eq16] represent pyrosulfate formation. Potassium pyrosulfate acts as an oxidizing acid, dissolving protective oxides (e.g., hematite, chromite) at subcritical temperatures (below 600 °C). This is particularly relevant in retrofit scenarios, where increased SO_3_ and HCl levels elevate the risk of corrosion. Mayoral et al.^117^ emphasize that trisulfate formation is a solid-phase hot corrosion mechanism, independent of melting, and propose it as the cause of metal loss observed in previous studies.^118,119^ eq 17 through 20 illustrate the formation of metallic trisulfates and the subsequent dissolution of protective scales.

15 $$2\left(\mathrm{Na},K\right){\mathrm{Cl}}_{\left(s\right)}{+\mathrm{2SO}}_{2(g)}+\frac{1}{2}O_{2(g)}{+H}_{2}O_{\left(g\right)}\to {\left(\mathrm{Na},K\right)}_{2}{\mathrm{SO}}_{4(s)}{+\mathrm{2HCl}}_{\left(g\right)}$$

16 $${\left(\mathrm{Na},K\right)}_{2}{\mathrm{SO}}_{4(s)}{+\mathrm{SO}}_{3(g)}\to {\left(\mathrm{Na},K\right)}_{2}S_{2}O_{7(s)}$$

17 $${3K}_{2}S_{2}O_{7(s)}{+\mathrm{Fe}}_{2}O_{3(s)}\to {2K}_{3}{\mathrm{Fe}(\mathrm{SO}}_{4}{)}_{3(s)}$$

18 $${3K}_{2}S_{2}O_{7(s)}{+\mathrm{Cr}}_{2}O_{3(s)}\to {2K}_{3}{\mathrm{Cr}(\mathrm{SO}}_{4}{)}_{3(s)}$$

19 $${2K}_{2}S_{2}O_{7(s)}{+\mathrm{2NiO}}_{\left(s\right)}\to K_{2}{\mathrm{Ni}}_{2}{{(\mathrm{SO}}_{4})}_{3\left(s\right)}{+K}_{2}{\mathrm{SO}}_{4(s)}$$

20 $${2K}_{2}S_{2}O_{7(s)}{+\mathrm{2FeO}}_{\left(s\right)}\to K_{2}{\mathrm{Fe}}_{2}{{(\mathrm{SO}}_{4})}_{3\left(s\right)}{+K}_{2}{\mathrm{SO}}_{4(s)}$$

Pyrosulfate formation is strictly related to molten-salt corrosion mechanisms. In high-sulfur environments, the dominant mechanism for low-temperature hot corrosion (LTHC) involves the formation of molten alkali–iron complex sulfates, which dissolve the protective oxide layer.^120^ eqs 21 and 22 detail the transformation of sulfates into pyrosulfates, which serve as precursors to alkali-iron sulfate salts. Following the destruction of the oxide scale, a corrosion cycle may develop wherein trisulfates react with the iron substrate to form iron sulfide (FeS), as described by eq 23.^121^

21 $${\mathrm{2SO}}_{2(g)}{+O}_{2(g)}\to {\mathrm{2SO}}_{3(g)}$$

22 $${\mathrm{SO}}_{3(g)}{+\mathrm{SO}}_{4(l)}^{\mathrm{2‐}}\to S_{2}O_{7(l)}^{\mathrm{2‐}}$$

23 $${\mathrm{9Fe}}_{\left(s\right)}{+K}_{3}{\mathrm{Fe}(\mathrm{SO}}_{4}{)}_{3(s)}\to {\mathrm{4Fe}}_{2}O_{3(s)}{+\mathrm{3FeS}}_{\left(g\right)}{+3K}_{2}{\mathrm{SO}}_{4(s)}$$

Table 4 presents the melting points of the selected pure salts and eutectic mixtures. It clearly illustrates the significant difference in melting behavior between pure compounds and their mixtures, thereby elucidating why hot corrosion can be initiated at relatively lower temperatures.

**Table 4:** Melting Points of Selected Pure Salts and Eutectic Mixtures

**Pure Salts**	**Melting points (°C)**	**Reference**
K_2_S_2_O_7_	325	[^30^]
KOH	360	[^111^]
KCl	772/776	
K_2_CO_3_	891	
K_2_CrO_4_	980	
K_2_SO_4_	1069	

**Eutectic mixtures**	**Melting points (°C)**	**Reference**
66 ZnCl_2_–34 FeCl_3_	200	[^110^]
68 ZnCl_2_–32 KCl	230	
51 ZnCl_2_–43 KCl – 6 PbCl_2_	238	
43 ZnCl_2_–49 KCl–8 PbCl_2_	250	
48 ZnCl_2_–52 KCl	250	
82 ZnCl_2_–18 KCl	262	
84 ZnCl_2_–16 KCl	262	
39 ZnCl_2_ – 50 KCl – 11 PbCl_2_	275	
73 ZnCl_2_–27 PbCl_2_	300	
9.3 K_2_CO_3_–90.7 KOH	366	[^111^]
25 KCl–75 K_2_Cr_2_O_7_	368	
17 ZnCl_2_–59 KCl – 34 PbCl_2_	375	[^110^]
21 KCl–79 PbCl_2_	411	
31 ZnCl_2_–69 KCl	430	
K_2_SO_4_–CoSO_4_	535	[^30^]
68.4 KCl–31.6 KCrO_4_	650	[^111^]
73.4 KCl–26.6 K_2_SO_4_	694	

The formation of eutectic mixtures is frequently the most critical factor in corrosive processes, as these substances exhibit the lowest melting points. Once liquefied on metallic surfaces, the diffusion of gaseous species toward the metal interface is significantly enhanced, thereby accelerating chemical reactions via the oxidation mechanism. In this context, Mahajan and Chibber^122^ demonstrated that the chromium content is the most decisive factor for hot corrosion resistance. High-chromium steels (such as SS304L) develop a protective chromium­(III) oxide (Cr_2_O_3_) scale. Furthermore, the authors evaluated the impact of environments rich in chlorine and vanadium­(V), showing that these species are highly detrimental to boiler materials, particularly those with low chromium levels. Steels such as P22 and P91, evaluated in their study, followed parabolic oxidation kinetics, indicating the formation of thick, nonprotective oxide scales.

A fundamental characteristic of all hot corrosion mechanisms is the destruction of the protective oxide scales. This phenomenon continuously exposes the underlying alloying elements to aggressive combustion gases. Consequently, the reactions describing the dissolution of the most common oxides follow the mechanisms presented in eq 24–29, corresponding to acidic and basic pathways, respectively.^30^ Figure 8 illustrates metal destruction due to oxidation mechanism by molten salts.

**Figure 8:**
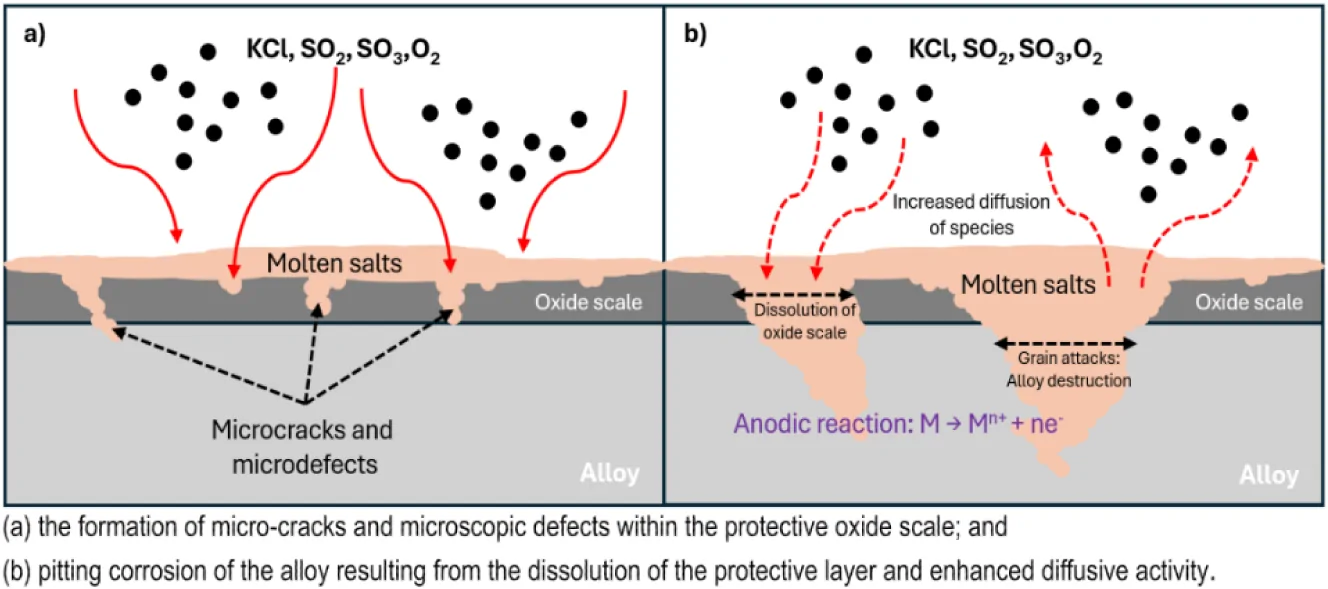
Schematic representation of hot corrosion via molten salt formation. Adapted from Journal of Materials Science and Technology, Vol. 90, S. Hu, H. Finklea, X. Liu, A review on molten sulfate salts induced hot corrosion, Pages 243–254, Copyright (2021), with permission from Elsevier.

• Acid pathway:,

24 $${\mathrm{Fe}}_{2}O_{3(s)}{+\mathrm{Na}}_{2}O_{\left(s\right)}⇌{\mathrm{2NaFeO}}_{2(s)}$$

25 $${\mathrm{4Fe}}_{3}O_{4(s)}{+\mathrm{6Na}}_{2}O_{\left(s\right)}{+O}_{2(g)}⇌{\mathrm{12NaFeO}}_{2(s)}$$

26 $${\mathrm{2Cr}}_{2}O_{3(s)}{+\mathrm{2Na}}_{2}O_{\left(s\right)}{+4O}_{2(g)}⇌{\mathrm{4NaCrO}}_{4(s)}$$

• Basic pathway:

27 $${\mathrm{Fe}}_{2}O_{3(s)}{+\mathrm{3Na}}_{2}{\mathrm{SO}}_{4(s)}⇌{\mathrm{Fe}}_{2}{{(\mathrm{SO}}_{4})}_{3\left(s\right)}{+\mathrm{3Na}}_{2}O_{\left(s\right)}$$

28 $${\mathrm{2Fe}}_{3}O_{4(s)}{+\mathrm{6Na}}_{2}{\mathrm{SO}}_{4(s)}⇌{\mathrm{6FeSO}}_{4(s)}{+\mathrm{6Na}}_{2}O_{\left(s\right)}{+O}_{2(g)}$$

29 $${\mathrm{Cr}}_{2}O_{3(s)}{+\mathrm{3Na}}_{2}{\mathrm{SO}}_{4(s)}⇌{\mathrm{Cr}}_{2}{{(\mathrm{SO}}_{4})}_{3}{+\mathrm{3Na}}_{2}O_{\left(s\right)}$$

The formation of low-melting-point eutectic mixtures involving KCl, NaCl, and contaminants such as ZnCl_2_ and PbCl_2_ (see Table 4) can also generate a liquid phase on tube surfaces at operational temperatures.^123^ The presence of these mixtures is highly relevant to this corrosion mechanism. Santos^110^ addressed the challenges posed by these contaminants during municipal solid waste (MSW) combustion; the vast heterogeneity of such fuels promotes the release of trace elements (Zn and Pb) in the form of chlorides. However, a scientific knowledge gap remains regarding the specific chemical transformations that these compounds undergo.

### 5.3 Corrosion Induced by Potassium Hydroxides and Carbonates

Compared with KCl and K_2_SO_4_, there is a noticeable gap in the scientific literature regarding the isolated corrosive effects of potassium carbonates and hydroxides in biomass combustion environments. Potassium carbonates and hydroxides are frequently underestimated, despite playing an equally destructive role in the degradation of steam generators.

Blomberg^21,42^ proposed that gaseous KOH acts as the primary initiator of corrosion in biomass-fired boilers. According to the suggested mechanism, excess potassium forms KOH, which condenses as a highly basic liquid film on superheater surfaces, thereby triggering corrosive attack, a hypothesis reinforced by electrochemical probe measurements that detected ionic activity (indicating molten electrolyte formation) at approximately 400 °C. This perspective establishes a new criterion for material selection: in environments governed by molten KOH films, chromium-rich alloys may exhibit inferior performance, making nickel-based superalloys more suitable options due to the superior stability of their oxides in basic media.

In a different analytical scope, Weng et al.^124^ developed an optical methodology for the online, quantitative measurement of key potassium species (KOH, KCl, and atomic K). Their study demonstrates that gaseous KOH is one of the primary potassium species present in flue gases, alongside gaseous KCl. While their relative proportions vary significantly depending on combustion conditions (oxygen-rich vs oxygen-lean), KOH consistently maintains significant concentrations. This research robustly validates prior theoretical premises, transitioning gaseous KOH from a theoretical possibility to a measurable and abundant reality in combustion environments. It provides the quantitative diagnostics necessary to transform hypotheses into experimentally proven facts, enabling future corrosion models to be supported by empirical data regarding aggressive agent concentrations. Potassium hydroxide is fundamentally relevant due to its high corrosivity at typical operating temperatures (400–600 °C) and its extreme basicity, which facilitates numerous reactions leading to the formation of the aforementioned corrosive compounds.^125^ The primary reactions in this context are presented in eq 30 through 33.

30 $${\mathrm{2KOH}}_{\left(g\right)}{+\mathrm{SO}}_{2(g)}+\frac{1}{2}O_{2(g)}⇌K_{2}{\mathrm{SO}}_{4(s)}{+H}_{2}O_{\left(g\right)}$$

31 $${\mathrm{KOH}}_{\left(l\right)}{+\mathrm{HCl}}_{\left(g\right)}\to {\mathrm{KCl}}_{\left(s\right)}{+H}_{2}O_{\left(g\right)}$$

32 $${\mathrm{2KOH}}_{\left(l,g\right)}{+\mathrm{SO}}_{3(g)}\to K_{2}{\mathrm{SO}}_{4(s)}{+H}_{2}O_{\left(g\right)}$$

33 $${\mathrm{2KOH}}_{\left(l,g\right)}{+\mathrm{CO}}_{2(g)}\to K_{2}{\mathrm{CO}}_{3\left(s\right)}{+H}_{2}O_{\left(g\right)}$$

Regarding K_2_CO_3_, its destructive effect is similar to the mechanisms previously described for KCl. Pettersson et al.^126^ investigated the aggressiveness of K_2_CO_3_ on higher-chromium stainless steels, reporting that it causes the depletion of the protective oxide scale within the 500–600 °C temperature range. Gravimetric analyses revealed that oxide production in the presence of K_2_CO_3_ was even greater than that observed with KCl within this temperature regime. This indicates that the reactions proceed with greater intensity at the oxide/metal interface due to the enhanced breakdown and dissolution of the protective oxide barrier. eqs 34 and 35 illustrate the dissolution reactions of these protective scales.

34 $$\frac{1}{2}{\mathrm{Cr}}_{2}O_{3\left(s\right)}{+\mathrm{2KCl}}_{\left(s\right)}{+H}_{2}O_{\left(g\right)}+\frac{3}{4}O_{2\left(g\right)}⇌K_{2}{\mathrm{CrO}}_{4\left(s\right)}{+\mathrm{2HCl}}_{\left(g\right)}$$

35 $$\frac{1}{2}{\mathrm{Cr}}_{2}O_{3(s)}{+K}_{2}{\mathrm{CO}}_{3(s)}+\frac{3}{4}O_{2(g)}⇌K_{2}{\mathrm{CrO}}_{4(s)}{+\mathrm{CO}}_{2(g)}$$

In contrast, the reactivity of K_2_CO_3_ with the passive oxide scale is addressed by Lehmusto et al.,^127,128^ who concluded that despite the deleterious effects of K_2_CO_3_, its aggressiveness is significantly lower than that of KCl. Both studies focus on corrosion in resistant alloys, specifically 304L stainless steel and the nickel-based superalloy 625. The results indicate a breakdown in the material’s passivity; however, the difference in aggressiveness is stark. While KCl causes severe corrosion in both materials, K_2_CO_3_ triggers a milder and more homogeneous oxidation, particularly in Alloy 625, which manages to maintain its protective chromium oxide scale. This comparison illustrates a fundamental principle in materials science and corrosion engineering: a substance cannot be judged as “corrosive” in isolation. Its behavior is critically environment-dependent (governed by temperature, phase, concentration, moisture, etc.). Therefore, evaluating the role of various chemical compounds across different systems requires an intrinsic understanding of the context in which the problem is situated.

In addition to the aforementioned mechanisms, the interaction between K_2_CO_3_ and water vapor is of significant concern, as their reaction promotes the formation of potassium hydroxide (KOH), which similarly drives corrosive degradation. A recent study by Lindmark et al.^129^ details the consequences of KOH generation in biomass combustion environments. Their research demonstrates that even trace amounts of gaseous KOH, as low as 16 ppm, drastically accelerate the corrosion of stainless steels and nickel-based superalloys, leading to the rapid destruction of the protective chromium oxide scale. Furthermore, the study identifies the formation of potassium ferrate (KFeO_2_) as a secondary corrosion product, suggesting that KOH attacks both the primary chromium-rich scale and the secondary iron oxide layer that develops following scale breakdown. eq 36 through 39 present the corrosive reactions described in their work.

36 $$K_{2}{\mathrm{CO}}_{3(s)}{+H}_{2}O_{\left(g\right)}⇌{\mathrm{2KOH}}_{\left(g\right)}{+\mathrm{CO}}_{2(g)}$$

37 $${\mathrm{4KOH}}_{\left(s,l,g\right)}{+\mathrm{Cr}}_{2}O_{3(s)}+\frac{1}{2}O_{2(g)}⇌{2K}_{2}{\mathrm{CrO}}_{4(s)}{+2H}_{2}O_{\left(l,g\right)}$$

38 $${\mathrm{Fe}}_{2}O_{3(s)}{+\mathrm{2KOH}}_{\left(s,l,g\right)}⇌{\mathrm{2KFeO}}_{2(s)}{+H}_{2}O_{\left(g\right)}$$

39 $${\mathrm{Fe}}_{3}O_{4(s)}{+\mathrm{3KOH}}_{\left(g\right)}+\frac{1}{4}O_{2(g)}⇌{\mathrm{3KFeO}}_{2(s)}+\frac{3}{2}H_{2}O_{\left(g\right)}$$

Figure 9 provides a schematic overview of the corrosion mechanism induced by these species, integrating the individual reactions detailed in this section. The process originates during biomass pyrolysis, when sulfur, chlorine, and potassium are released in excess into the flue gas. Gaseous potassium subsequently condenses on cooler superheater surfaces as a highly basic KOH liquid film that reacts with the surrounding gas environment. At the metal interface, this condensed phase drives the destruction of the protective oxide scale: KOH and K_2_CO_3_ dissolve the chromium-rich Cr_2_O_3_ layer to form soluble potassium chromate (K_2_CrO_4_, eqs 35 and 37), while KOH further attacks the underlying iron oxide layer to yield potassium ferrate (KFeO_2_, eqs 38 and 39).

**Figure 9:**
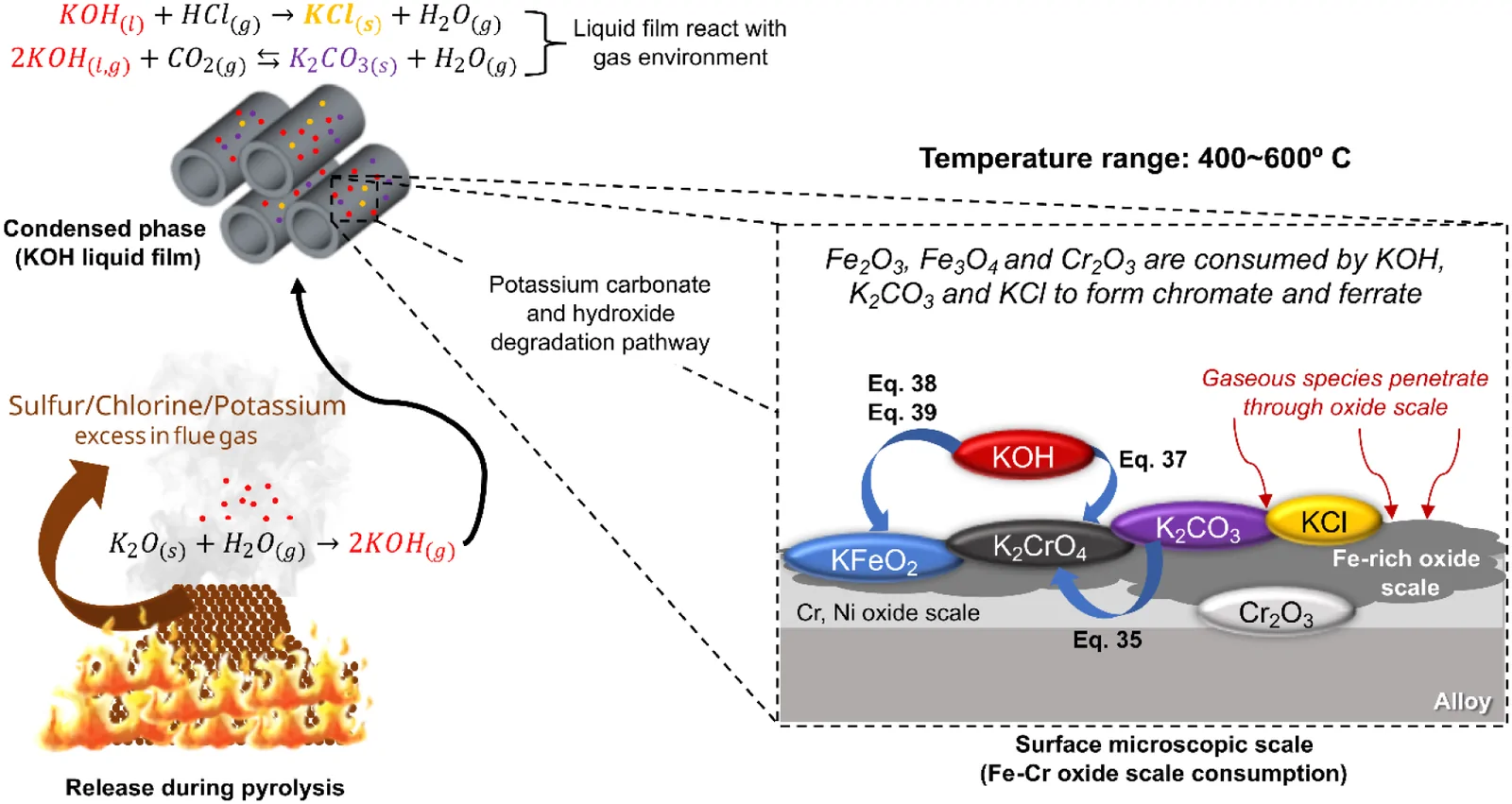
Schematic representation of the corrosion mechanism induced by potassium hydroxide and carbonate.

### 5.4 The Ambivalent Role of Sulfur

Sulfur, which is less abundant in biomass compared to coal, plays a dual and complex role within the boiler environment. Its behavior at high temperatures is closely interrelated with the presence of alkali salts. The duality of sulfur’s effects exposes one of the most controversial aspects of this field and represents a crucial leverage point for corrosion control. It is well established that during the combustion process, sulfur is present in the flue gas primarily in two forms: SO_2_ and SO_3_. In the absence of sufficient sulfur, KCl-induced corrosion becomes the dominant and devastating mechanism.^129^ Hindiyarti et al.^131^ conducted an exploratory study presenting various kinetic models to describe the phenomenon of alkali aerosol formation during biomass combustion. The study discusses homogeneous gas-phase nucleation, which involves several conversion steps of stable and unstable compounds. Consequently, it concludes that the rate-limiting step for K_2_SO_4_ production is the oxidation of SO_2_ to SO_3_.

Figure 10 summarizes this ambivalent behavior, which is ultimately governed by the fuel’s Cl/S ratio. Along the beneficial pathway, favored at high Cl/S ratios, sulfur acts to sequester chlorine-bound alkalis: it sulfates highly corrosive, low-melting KCl into the far more stable, high-melting-point K_2_SO_4_. Conversely, along the deleterious pathway, favored at low Cl/S ratios, excess sulfur becomes aggressive: the oxidation of SO_2_ to SO_3_ supplies the SO_3_ required to form corrosive alkali-iron trisulfates and pyrosulfates (K_3_Fe­(SO_4_)_3_) and K_2_S_2_O_7_, which dissolve the protective oxide scale. The following paragraphs examine each of these opposing roles and the conditions that determine which prevails.

**Figure 10:**
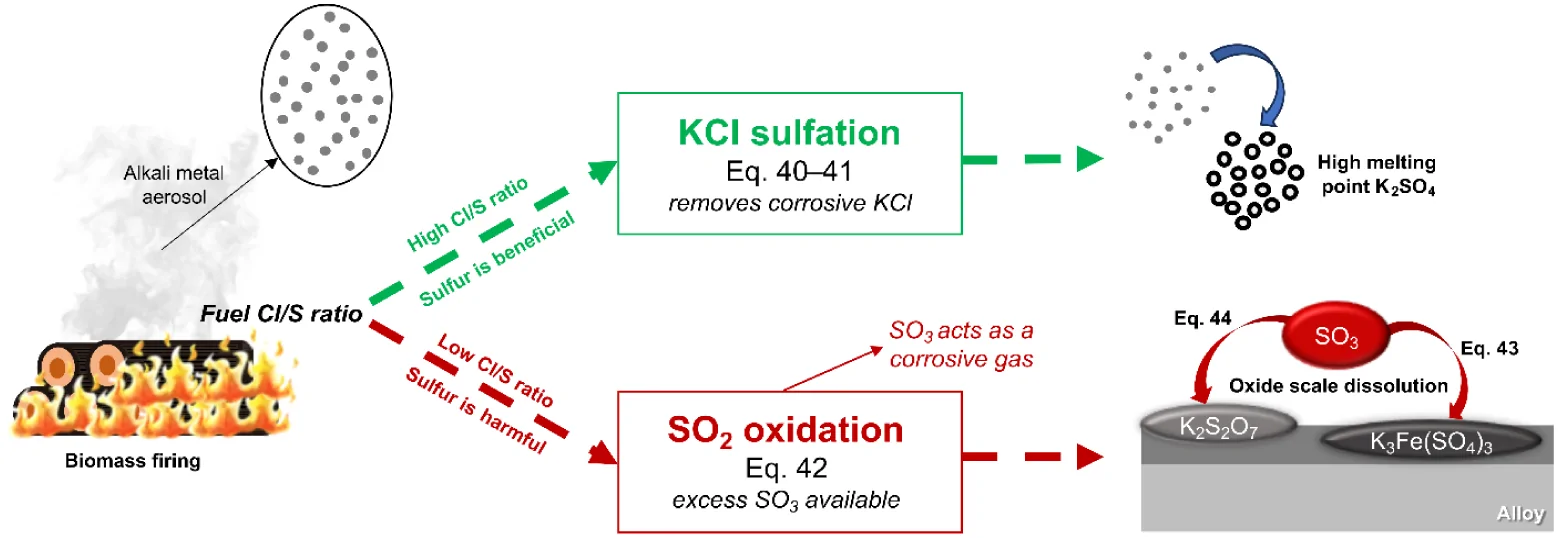
Schematic representation of the ambivalent role of sulfur in high-temperature corrosion, governed by the fuel Cl/S ratio.

The relevance of the KCl sulfation reaction has been the focus of several studies and represents the beneficial effect of sulfur in mitigating corrosion. eqs 40 and 41 present the sulfation reactions that convert KCl, which is highly corrosive and has a low melting point, into K_2_SO_4_,^130,132^ a compound that is chemically more stable, significantly less aggressive, and possesses a considerably higher melting point (1069 °C).^133^ However, this reaction presents a critical caveat. The generation of Cl_2_ and HCl near the gas/oxide interface can promote further degradation via the active oxidation mechanism.

40 $${\mathrm{2KCl}}_{\left(s\right)}{+\mathrm{SO}}_{2(g)}{+O}_{2(g)}\to K_{2}{\mathrm{SO}}_{4(s)}{+\mathrm{Cl}}_{2(g)}$$

41 $${\mathrm{2KCl}}_{\left(g\right)}{+\mathrm{SO}}_{2(g)}+\frac{1}{2}O_{2(g)}{+H}_{2}O_{\left(g\right)}\to K_{2}{\mathrm{SO}}_{4(s)}{+\mathrm{2HCl}}_{\left(g\right)}$$

The positive impact of sulfur further extends to the capture of fine particles in combustion gases. The work of Lind et al.^134^ represents a significant advancement in this area, as it highlights that beneficial actions initiate prior to deposit formation. Sulfur acts as a “cleaning agent” in the gas phase, scavenging volatile alkalis to form more stable and less problematic sulfate compounds, rather than allowing them to form sticky KCl-rich fine particles. The authors suggest that this may occur through the formation of mixed K–Ca sulfate particles, which are coarser and less prone to forming dense, adherent deposits on superheaters. Furthermore, an additional beneficial effect was identified by Yin and Wu,^135^ who discussed how SO_2_ can promote an extra protective layer at the gas/oxide interface through the formation of iron sulfate (FeSO_4_). This product acts as a diffusion barrier, effectively hindering chlorine access to the steel substrate.

In contrast, the deleterious role of sulfur involves the formation of critical compounds on metal surfaces during the cofiring of biomass with coal or with biomass/waste blends having high sulfur content. Hussain et al.^119^ compared air-firing with oxy-combustion, where SO_*x*_ concentrations are significantly higher. They observed that higher SO_*x*_ levels in oxy-combustion increase the peak corrosion temperature (from 650 to 700 °C). This occurs because excess SO_3_ stabilizes corrosive trisulfates at higher temperatures, broadening the window of severe attack. In laboratory tests, Holcomb et al.^136^ also found that increasing SO_2_ concentration in the simulated gas significantly raised corrosion rates for various alloys at their peak temperatures. Certain harmful reactions involving sulfur compounds were addressed by Li and Zeng,^31^ who described this specific mechanism. eq 42 –44 present these reactions.

42 $${\mathrm{SO}}_{2(g)}+\frac{1}{2}O_{2(g)}\to {\mathrm{SO}}_{3(g)}$$

43 $${3K}_{2}{\mathrm{SO}}_{4(s)}{+\mathrm{Fe}}_{2}O_{3(s)}{+\mathrm{3SO}}_{3(g)}\to {2K}_{3}{\mathrm{Fe}(\mathrm{SO}}_{4}{)}_{3(s,l)}$$

44 $${\mathrm{SO}}_{3(g)}{+K}_{2}{\mathrm{SO}}_{4(s)}\to K_{2}S_{2}O_{7(s,l)}$$

The defining factor for sulfur’s role in high-temperature corrosion is the Cl/S ratio. In systems with a high ratio, typical of agricultural biomass such as straw, husks, and seeds, chlorine in conjunction with alkali metals acts as the primary aggressive agent; in these cases, sulfur serves as a mitigator of destructive mechanisms. Conversely, when the Cl/S ratio is low, the role of sulfur compounds is inverted, and excess sulfur itself becomes a threat to material integrity by intensifying the prevailing corrosion mechanisms. Modern boiler management entails more than just selecting resistant alloys; it requires an intrinsic understanding and, where feasible, the manipulation of fuel chemistry. For chlorine-rich biomass, this necessitates ensuring sufficient sulfur availability through cofiring or additives to counteract the deleterious effects of chlorine.

## 6 Mitigation Strategies

Several common approaches to preventing corrosive attack discussed in the literature involve interventions that can be classified as either proactive or reactive, depending on the targeted outcome. However, many of these mitigation strategies present significant challenges for direct implementation, whether due to associated economic costs or practical feasibility concerning the design and operation of industrial boilers.

### 6.1 Fuel Management

Li et al.^137^ experimentally investigated the cocombustion of municipal sewage sludge (MSS) with wheat straw, revealing a significant shift in ash chemistry and potentially critical potassium retention. X-ray diffraction (XRD) analyses indicated the formation of high-melting-point aluminosilicate compounds (KAlSi_3_O_8_ and KAlSi_2_O_6_) that are not present when the pure fuels are fired.

Matúš et al.^138^ focused on producing high-quality pellets for small-scale boilers using various proportions of wheat straw and papermaking sludge. Their results demonstrated a substantial increase in the ash melting point; notably, with 20% or more sludge in the pellet composition, no evidence of sintering or slagging was observed.

Subsequently, Pan et al.^139^ evaluated the introduction of a sludge-derived modifying agent, composed primarily of calcium phosphate in concentrations ranging from 2.5% to 7.5% alongside rice husk. This approach resulted in a significant enhancement of potassium retention in the residual bottom ash. Furthermore, the additive improved the thermal properties of the ash, elevating its melting point by up to 238 °C (from 1214 to 1452 °C) when applied at a 5.0% ratio. Similarly, Wang et al.^140^ assessed the cocombustion of sewage sludge and rice husk to mitigate challenges related to deposit formation and fouling. They also observed an increase in ash fusion temperature, leading to a remarkable reduction in slagging, particularly at higher sludge concentrations.

The cocombustion of plastic waste with biomass has also been investigated. Karuana et al.^141^ evaluated corrosion effects in systems cofiring mixtures of plastic, biomass, and coal. These mixtures were categorically classified into low, medium, high, and severe risk groups based on alkali metal concentrations. eqs 45 and 46 estimate the corrosive potential (φ) of these mixtures, and Table 5 summarizes the categories and corrosive potential derived from these equations, as proposed by the authors. The findings indicated that a 25% blend of biomass and plastic with coal is unviable, as it accelerates corrosion. The resistance of austenitic steel was compromised, primarily due to the degradation of the protective Cr_2_O_3_ scale.

45 $$\varphi =\frac{\mathrm{Na}+\mathrm{K - 2S - Cl}}{\mathrm{PCI}}$$

46 $$\varphi =\frac{\mathrm{Na}+K}{2S+\mathrm{Cl}}$$

**Table 5:** Corrosion Risk Based on the Concentration of Critical Species in Biomass Ash^a^

**Potential:**	**Low**	**Mid**	**High**	**Severe**
% Cl	<0,3	0,3 < 0,5	>0,5	–
% S/Cl	>4,0	2,0∼4,0	<2,0	–
eq 45 (φ)	<0,004	0,004∼0,007	0,007∼0,010	>0,010
eq 46 (φ)	>1: indicate the excess of alkalis produced			
	<1: indicates high HCl and SO_*x*_ emission in the gas phase			

a Adapted from Engineering Failure Analysis, Vol. 150: F. Karuana, A. Prismantoko, N. Suhendra, A. Darmawan, H. Hariana, D. B. Darmadi, M. A. Muflikhun, Investigation of austenitic stainless steel corrosion resistance against ash deposits from co-combustion coal and biomass waste, 107368, Copyright (2023), with permission from Elsevier.

In a recent study, Zhao et al.^142^ departed from traditional single-factor investigations to focus on the coupled interaction between ash deposition and acidic gases (HCl and SO_2_). Their evaluation of corn stalk and lignite coal cocombustion revealed that corrosion is significantly more pronounced under the biomass-coal mixture. This research is particularly noteworthy as it demonstrates that isolated testing methodologies (evaluating only gas or only ash) dangerously overestimate the lifespan of boiler components. The finding that mixed ash can be more corrosive than pure biomass ash directly challenges the prevailing assumption that coal can “dilute” biomass corrosivity via alkali capture by aluminosilicates. Consequently, it is imperative to evaluate mitigation strategies through a systemic, multifactor approach that accounts for ash composition, gaseous constituents, and the metallic interface. Simply blending fuels can be detrimental unless the approach incorporates precise adjustments to the biomass ratio alongside additives with the appropriate chemical profile.

Leaching also constitutes a pretreatment alternative that yields good practical results. The primary objective of this method is to remove harmful salts associated with corrosion by “washing” the fuel with solvents that possess physicochemical affinities. Liu et al.^143^ provided an objective comparison between deionized water, nitric acid (HNO_3_), and phosphoric acid (H_3_PO_4_) in the washing of rice straw. The results for ash removal between deionized water and HNO_3_ at a concentration of 2.0 kmol m^–3^ were similar, reaching approximately 10% removal. Furthermore, removal using H_3_PO_4_ at 0.5 kmol m^–3^ proved to be the most effective, eliminating up to 93% of alkali metals; conversely, it drastically delayed char combustion as a consequence of the activation of insoluble alkalis bound to the organic matrix.

Carrillo et al.^144^ identified direct consequences of washing sorghum biomass with water. While the contents of magnesium, nitrogen, calcium, potassium, and chlorine decreased, the concentrations of hydrogen, carbon, iron, aluminum, silica, titanium, sodium, and sulfur significantly increased—most notably lignin content. The ash content was reduced by up to 20%, whereas lignin increased by 53%. This study, alongside previous research, highlights the undesirable implications of this method regarding fuel performance during combustion. The lignin enrichment increases the fixed carbon (char) fraction and reduces the volatile release rate, necessitating a longer residence time for complete combustion. This potentially compromises flame stability in boilers designed for the rapid-combustion profile of the raw biomass.

Water is the primary solvent used in biomass leaching processes across various studies, frequently contributing to positive outcomes regarding ash reduction.^145–148^ Furthermore, Yu et al.^149^ investigated the impact of water washing on several different biomass types; their results indicated a more complex outcome, involving a decrease in both ash and organic matter content. In addition, one of the most notable improvements was the elevation of ash fusion temperatures, which in some instances exceeded 500 °C. However, despite yielding a leached product with essential characteristics for mitigating corrosive issues, the process results in biomass with a remarkably high moisture content, thereby necessitating subsequent drying.

Guo et al.^150^ utilized aqueous and dilute HCl solutions for the leaching of corn straw; their results showed an increase in hydrogen and carbon contents, while nitrogen and sulfur levels decreased. The ash content in the acidified leachate was higher compared to that of water washing, and both treatments resulted in an increase in volatile matter, fixed carbon, and the biomass heating value. The HCl-based acid leaching employed in this study influenced the fuel’s thermal performance differently than the findings of Liu et al.,^143^ thereby opening new avenues for discussion regarding solvent-specific effects on combustion kinetics.

### 6.2 Prevention through Additive Management

Solutions aimed at mitigating corrosion processes without fuel pretreatment are generally more complex and are often referred to as in situ management. This approach typically involves the application of compounds during combustion, frequently via separate injection systems in large-scale plants, such as fluidized beds. Additives may be injected independently into the combustion chamber to react with released alkali vapors. The primary strategy in this regard is the addition of small quantities of additives, generally totaling less than 5% of the total mass. In this context, kaolin (Al_2_Si_2_O_5_(OH_4_)) and metakaolin are notable for frequently yielding positive results in altering the ash fusion temperature and providing thermal stabilization.^151,152^ The capture of KCl and K_2_SO_4_ can be simplified by eq 47 through 50.

47 $${\mathrm{Al}}_{2}{\mathrm{Si}}_{2}O_{5}{\left(\mathrm{OH}\right)}_{4(s)}{+\mathrm{2KCl}}_{\left(s,g\right)}\to {\mathrm{2KAlSiO}}_{4(s)}{+H}_{2}O_{\left(g\right)}{+\mathrm{2HCl}}_{\left(g\right)}$$

48 $${\mathrm{Al}}_{2}{\mathrm{Si}}_{2}O_{5}{\left(\mathrm{OH}\right)}_{4(s)}{+\mathrm{2KCl}}_{\left(s,g\right)}{+\mathrm{2SiO}}_{2(s)}\to {\mathrm{2KAlSi}}_{2}O_{6(s)}{+H}_{2}O_{\left(g\right)}{+\mathrm{2HCl}}_{\left(g\right)}$$

49 $${\mathrm{Al}}_{2}{\mathrm{Si}}_{2}O_{5}{\left(\mathrm{OH}\right)}_{4(s)}{+K}_{2}{\mathrm{SO}}_{4(s)}\to {\mathrm{2KAlSiO}}_{4(s)}{+2H}_{2}O_{\left(g\right)}{+\mathrm{SO}}_{3(g)}$$

50 $${\mathrm{Al}}_{2}{\mathrm{Si}}_{2}O_{5}{\left(\mathrm{OH}\right)}_{4(s)}{+K}_{2}{\mathrm{SO}}_{4(s)}{+\mathrm{2SiO}}_{2(s)}\to {\mathrm{2KAlSi}}_{2}O_{6(s)}{+2H}_{2}O_{\left(g\right)}{+\mathrm{SO}}_{3(g)}$$

In these equations, the scavenging agent (kaolin or kaolinite) is added during combustion, yielding kalsilite (KAlSiO_4_) and leucite (KAlSi_2_O_6_) as products. These compounds are thermally stable and generally do not melt at boiler operating temperatures;^15^ their melting points exceed 1500 °C, which are considerably higher than those of pure biomass ashes. Alternatively, Wang et al.^153^ demonstrated that zeolites can also act as scavenging agents in addition to kaolin. Their results indicated that zeolite 24A significantly enhances the sintering characteristics of agricultural biomass ash, whereas zeolite Y exhibits low reactivity with potassium compounds, suggesting that not all zeolites are equally beneficial for this purpose. However, Wang et al.^154^ evaluated the effectiveness of these same additives during barley straw combustion and found that alkali metal capture is hindered by exposure time during boiler operation. This prolonged exposure reduces the reactivity of the mitigating agents toward these salts, compromising their effectiveness. Their study suggests that the effects of these additives should be evaluated with caution, as they may behave differently under varying operating conditions.

Wang et al.^155^ investigated the behavior of briquettes produced with phosphate-based additives, given the high reactivity these materials exhibit toward alkali salts. The combination of monoammonium phosphate (NH_4_H_2_PO_4_) and monocalcium phosphate (Ca­(H_2_PO_4_)_2_) with maize straw ash elevated the ash fusion temperature of the biomass briquettes by 55 to 277 °C. This stabilization results from the formation of phosphate products such as K_2_CaP_2_O_7_, K_2_CaP_4_O_12_, K_4_Ca­(PO_4_)_2_ and KCaPO_4_, which possess melting points ranging from 1195 to 1400 °C. eq 51 through 59 outline the mechanism of alkali salt capture by the phosphate additives explored in their study.

51 $${\mathrm{nNH}}_{4}H_{2}{\mathrm{PO}}_{4}+\mathrm{nKCl}\to {(\mathrm{KPO}}_{3}{)}_{n}{+\mathrm{nNH}}_{4}{\mathrm{Cl}+\mathrm{nH}}_{2}O$$

52 $${\mathrm{2NH}}_{4}H_{2}{\mathrm{PO}}_{4}+\mathrm{2KCl}+\mathrm{CaO}\to K_{2}{\mathrm{CaP}}_{2}O_{7}{+\mathrm{2NH}}_{4}{\mathrm{Cl}+2H}_{2}O$$

53 $${\mathrm{2NH}}_{4}H_{2}{\mathrm{PO}}_{4}{+K}_{2}O+\mathrm{CaO}\to K_{2}{\mathrm{CaP}}_{4}O_{12}{+\mathrm{4NH}}_{3}{+6H}_{2}O$$

54 $${\mathrm{Ca}(H}_{2}{\mathrm{PO}}_{4}{)}_{2}+\mathrm{CaO}\to {\mathrm{2CaHPO}}_{4}{+H}_{2}O$$

55 $${\mathrm{2CaHPO}}_{4}\to {\mathrm{Ca}}_{2}P_{2}O_{7}{+H}_{2}O$$

56 $${\mathrm{Ca}(H}_{2}{\mathrm{PO}}_{4}{)}_{2}+\mathrm{2KCl}+\mathrm{CaO}\to K_{2}{\mathrm{CaP}}_{2}O_{7}{+\mathrm{CaCl}}_{2}{+2H}_{2}O$$

57 $${\mathrm{Ca}(H}_{2}{\mathrm{PO}}_{4}{)}_{2}+\mathrm{2KCl}+\mathrm{2CaO}\to {\mathrm{2KCaPO}}_{4}+{\mathrm{CaCl}}_{2}{+2H}_{2}O$$

58 $${\mathrm{2Ca}(H}_{2}{\mathrm{PO}}_{4}{)}_{2}+\mathrm{2KCl}\to K_{2}{\mathrm{CaP}}_{4}O_{12}{+\mathrm{CaCl}}_{2}{+4H}_{2}O$$

59 $${\mathrm{2Ca}(H}_{2}{\mathrm{PO}}_{4}{)}_{2}+\mathrm{4KCl}+\mathrm{2CaO}\to K_{2}{\mathrm{Ca}(\mathrm{PO}}_{4}{)}_{2}{+\mathrm{2CaCl}}_{2}{+2H}_{2}O$$

### 6.3 Materials Management

In recent years, mitigation techniques focused on the metallurgical industry have gained prominence. Research highlights the development of corrosion-resistant metallic materials for the construction of boilers and steam generators. Sharma et al.^18^ presented two main approaches in this regard: the utilization of superalloys and the application of thermal coatings. Materials that stood out for exhibiting excellent corrosion resistance included Super 304H, Superni 718, Superni 76, and Superfer 800H, as they possess higher concentrations of nickel and chromium in their composition, resulting in highly protective oxide scales. Figure 11 presents a compilation of information regarding several alloys selected from the study. It is observed that alloy performance varies significantly depending on the applied thermal coating, such as Superni 75, Superni 76, and Superfer 800. The concentration of protective elements, specifically Cr and Ni, facilitates robust anticorrosive mechanisms (see Figure 11).

**Figure 11:**
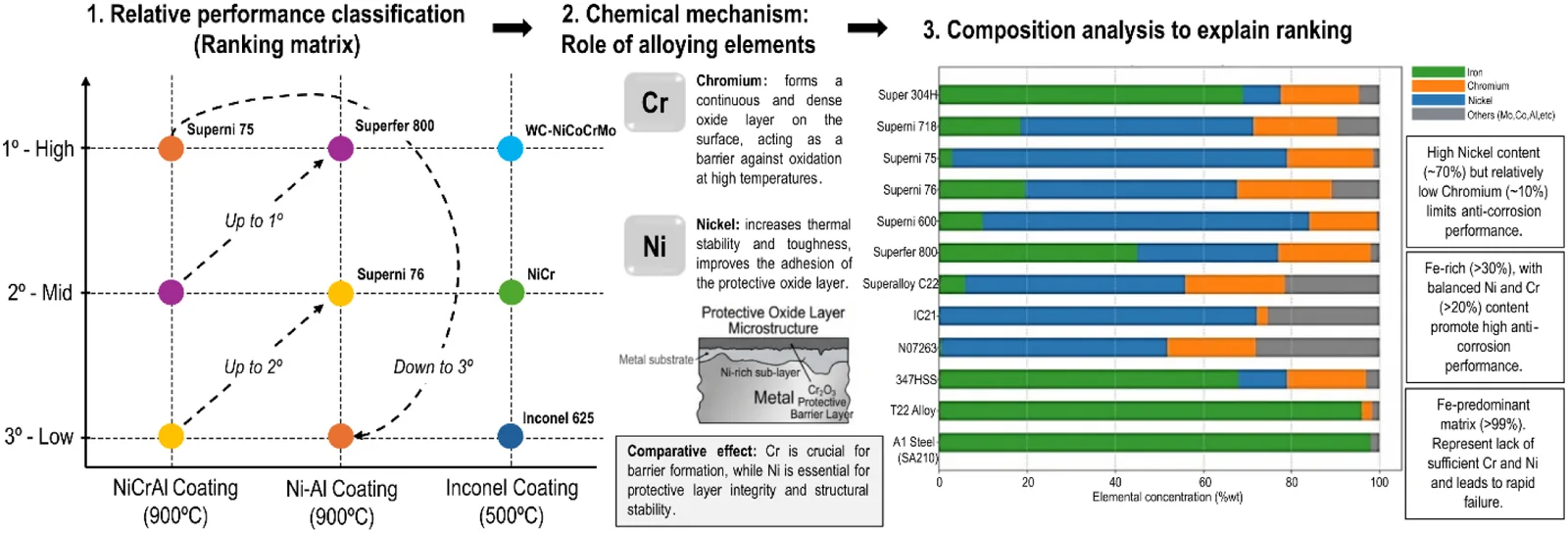
Performance anticorrosion ranking of super alloys.

Wu et al.^93^ discussed various strategies for thermal coatings on tubes, encompassing techniques such as HVOF, HVAF, laser cladding, and aluminization, which provide Ni- and Fe-based protection. The reported superiority of NiAl over NiCr is conditional rather than general. Wu et al.^93^ attribute the advantage of aluminide systems to the formation of a continuous Al_2_O_3_ barrier, and aluminizing indeed yields low porosity and strong metallurgical adhesion. However, Khantisopon et al.^156^ show that this advantage collapses in chlorine-bearing deposits unless a protective scale is pre-established: as-sprayed NiAl exposed to KCl at 600 °C allowed Cl_2_ diffusion to the metal surface, forming NiCl_2_ that subsequently oxidized into nonprotective NiO, whereas the same coating preoxidized at 700 °C for 6 h developed NiO along the splat boundaries and delayed Cl_2_ ingress. More critically, a diffusion-slurry aluminide on P91 steel failed outright under KCl at 560 °C after 336 h, with Al and Fe migrating toward the coating surface, leaving residual porosity into which KCl penetrated. Aluminide coatings should therefore not be recommended for chloride-rich biomass environments without prior oxide conditioning, a caveat that is systematically absent from the coating literature validated in sulfate-vanadate melts.

Zhang et al.^157^ suggested considering fundamental aspects to ensure the viability of coating adoption, ranging from the characteristics of the corrosion phenomenon to the refinement of analytical procedures and detailed cost evaluations. Small-scale experiments indicate that HVOF and cold spray techniques are the most prevalent, with a significant emphasis on NiCr alloys.

### 6.4 Protective Coatings and Claddings

Surface engineering decouples the corrosion problem from the fuel problem, imposing no constraint on feedstock supply, moisture, or combustion kinetics, decisive for plants under continuous steam demand. Some recent contributions, however, qualify the picture presented in Section 6.3 on four specific points:

I) Process, not composition, defines the corrosion path. Zhang et al.^157^ report HVOF and cold spray as the most prevalent techniques, but prevalence is not performance: Khantisopon et al.^156^ show that cold-sprayed NiCr, despite a denser microstructure than its HVOF counterpart, performed worse in an operating boiler because the lower process temperature produced a discontinuous Cr_2_O_3_ layer. Bermejo et al.^158^ observe the analogue in fusion cladding: overlay-welded FeCrAl EF100 suffered intergranular corrosion, whereas nonwelded specimens of the same alloy showed only a layered attack after 12 months in the field. Both results follow from the mechanism of Section 5.1: a single percolating path for Cl_2_ sustains the catalytic loop, and imply that specifications reporting only chemistry and porosity cannot predict boiler performance. Consistently, 1–2 wt % carbon nanotubes in Cr_3_C_2_–20NiCr reduced porosity from 1.85% to 1.74% yet cut corrosion rates by up to 82.96%.^156^
II) The alloy hierarchy of Figure 11 orders performance by Ni and Cr content. Field exposures invert this. In a 75 MW waste-fired CFB boiler under an alkali-chloride deposit with HCl, overlay-welded FeCrAl APMT matched the nickel-based reference Alloy 625 after 6 months, and an earlier campaign in the same plant gave 0.6 mm year^–1^ for APMT against 3.3 mm year^–1^ for the nickel-based Haynes HR-160. Iron-based HVOF Fe-27Cr likewise withstood 2 years in a biomass-fired boiler at 520–800 °C without chlorine ingress. Two consequences follow: the economic barrier lies in the nickel feedstock rather than in the deposition process (Section 8), and FeCrAl is a set of formulations rather than a performance class.^159^ Notably, the α-Al_2_O_3_ scale conventionally credited for FeCrAl forms readily only above 900 °C; at the tube temperatures of this exposure (∼310–330 °C) Cl was detected at the metal–oxide interface of all four alloys.
III) The reported performance of a coating is inseparable from the electrochemical configuration in which it was measured. Xu et al.^160^ clad Ni60 on one face of 316L and laser-remelted the other, exposing the assembly to molten NaCl-MgCl_2_–KCl at 700 °C: the coating suffered only 45 μm of attack after 1200 h against >90 μm for the remelted face, but the pair formed a macro-galvanic couple in which the coating was the cathode. Their own isolated specimens settle the point: a symmetrically Ni-coated sample reached 34.3 μm after 100 h, exceeding the 29 μm recorded at 400 h in the couple and the authors concede that the coupled depth is not a standalone service limit. Zhu et al.^159^ make the dependence explicit by design, coupling HT700 to an Al-based sacrificial anode and cutting corrosion depth by 75.64%; the benefit, however, is kinetic and consumable, decaying as the Al-enriched layer loses continuity. Neither case alters the corrosion path: in Xu’s couple, Cr dissolved preferentially, and Mg_2_SiO_4_ grew on both surfaces, so the percolation argument of Section 5.1 holds regardless of applied potential. Since weld overlays and dissimilar-material transitions are precisely the geometries reported by Bermejo et al.^158^ that embed couples of unknown signs into the tube wall, a rate measured in one configuration does not transfer to another.
IV) Corrosion metrics based on scale thickness also ignore the mechanical state of the substrate. Li et al.^161^ precorroded the single-crystal superalloy DD6 in NaCl-Na_2_SO_4_ at 850 °C and then cycled it: layers of only 18.6–22.6 μm under 1.2% of specimen thickness cut low-cycle fatigue life by more than an order of magnitude, requiring an exponent of 16.997 on the normalized layer thickness in their modified Basquin fit. Crack initiation migrated from internal casting defects to surface pits and oxide spikes bearing sulfide inclusions, the γ′-depleted layer beneath the Al_2_O_3_ scale removed load-bearing capacity, and ratcheting strain spalled the scale, re-exposing bare metal to the melt. The protective scale is thus simultaneously the site of substrate depletion, which explains why porosity and mass-gain data alone, as argued in point 1, cannot qualify a coating for a tube subject to thermal cycling and soot-blowing. One caveat bounds this reading: none of these environments is that of a biomass grate boiler; Xu et al.^160^ and Zhu et al.^159^ address chloride storage melts under argon or vacuum, where MgCl_2_ hydrolysis rather than KCl deposition drives the attack; and Li et al.^161^ a sulfate deposit on a cast superalloy under stresses irrelevant to superheater service. Their value is methodological, identifying two variables: galvanic configuration and mechanical loading that the biomass coating literature reports almost exclusively under isothermal, electrochemically undefined, unstressed conditions.

### 6.5 Comparative Assessment of Mitigation Strategies

Table 6 makes explicit a trade-off that is otherwise implicit throughout the literature: the strategies with the highest industrial readiness (fuel blending, kaolin dosing, Ni-based HVOF coatings) are those that either constrain fuel supply or import cost, whereas the strategies with the most favorable cost-effectiveness ratio at laboratory scale have not been validated under KCl-dominated deposits in operating boilers. No single strategy is simultaneously effective, inexpensive, and mature, which explains why industrial practice converges on combinations, typically additive dosing under continuous operation plus selective coating of the highest-flux superheater passes. Two consequences follow for the Brazilian context (Section 8): the least mature options are also the least import-dependent, and the readiness gap is concentrated in qualification and standardization rather than in fundamental science.

**Table 6:** Summary of Mitigation Strategies and Your Respective Limitations

**Strategy**	**Mechanism**	**Key Limitation**	**Cost**
Co-combustion/Fuel blending	Alkali capture by aluminosilicates	Mixed ash may be more corrosive than pure biomass ash; requires steady cofuel supply	Low
Leaching (water)	Removal of soluble K, Cl	High residual moisture → drying penalty; lignin +53% alters combustion profile	Low
Leaching (acid)	Removal of bound alkali	Delays char burnout; effluent management	Medium
Kaolin/Metakaolin additives	KCl → KAlSiO_4_, KAlSi_2_O_6_	Reactivity decays with exposure time; not all zeolites effective	Medium
Phosphate additives	K_2_CaP_2_O_7_, KCaPO_4_	Cost at industrial scale; Phosphorus recycling	Medium
Ni-based superalloys	Cr_2_O_3_/NiO scale	Capital cost; not economical for full retubing	Very High
FeCrAl/Fe–Cr alloys and coatings	Al_2_O_3_/Cr_2_O_3_ scale	Limited data under KCl vs Na_2_SO_4_–V_2_O_5_	Medium
HVOF NiCr/Cr_3_C_2_–NiCr	Continuous Cr_2_O_3_+dense splat structure	Ni feedstock cost; on-site application on tube banks	High
Cold spray NiCr	Solid-state deposition	Discontinuous Cr_2_O_3_ → inferior to HVOF	Medium
Laser cladding/EHLA	Metallurgical bond, grain refinement	Conventional <1 m.min^–1^; EHLA assessed mostly electrochemically	Very High
Carbide-reinforced coatings	Interruption of percolating diffusion paths	Feedstock cost; no long-term field data	Very High
Antislagging coatings	Reduced wettability of molten ash	No standard test method; slurry spray route slow	Medium

## 7 State of Art of Experimental Studies to Evaluated High-Temperature Corrosion

This section presents a compilation of experimental studies investigating phenomena that were not addressed in detail in previous sections. Table 7 summarizes the methodologies employed to study a wide range of biomass feedstocks under various corrosive environments. Furthermore, Figure 12 illustrates a comprehensive four-phase framework, synthesized from the relevant literature, for the investigation and mitigation of ash deposition and corrosion in biomass-fired boilers.

**Figure 12:**
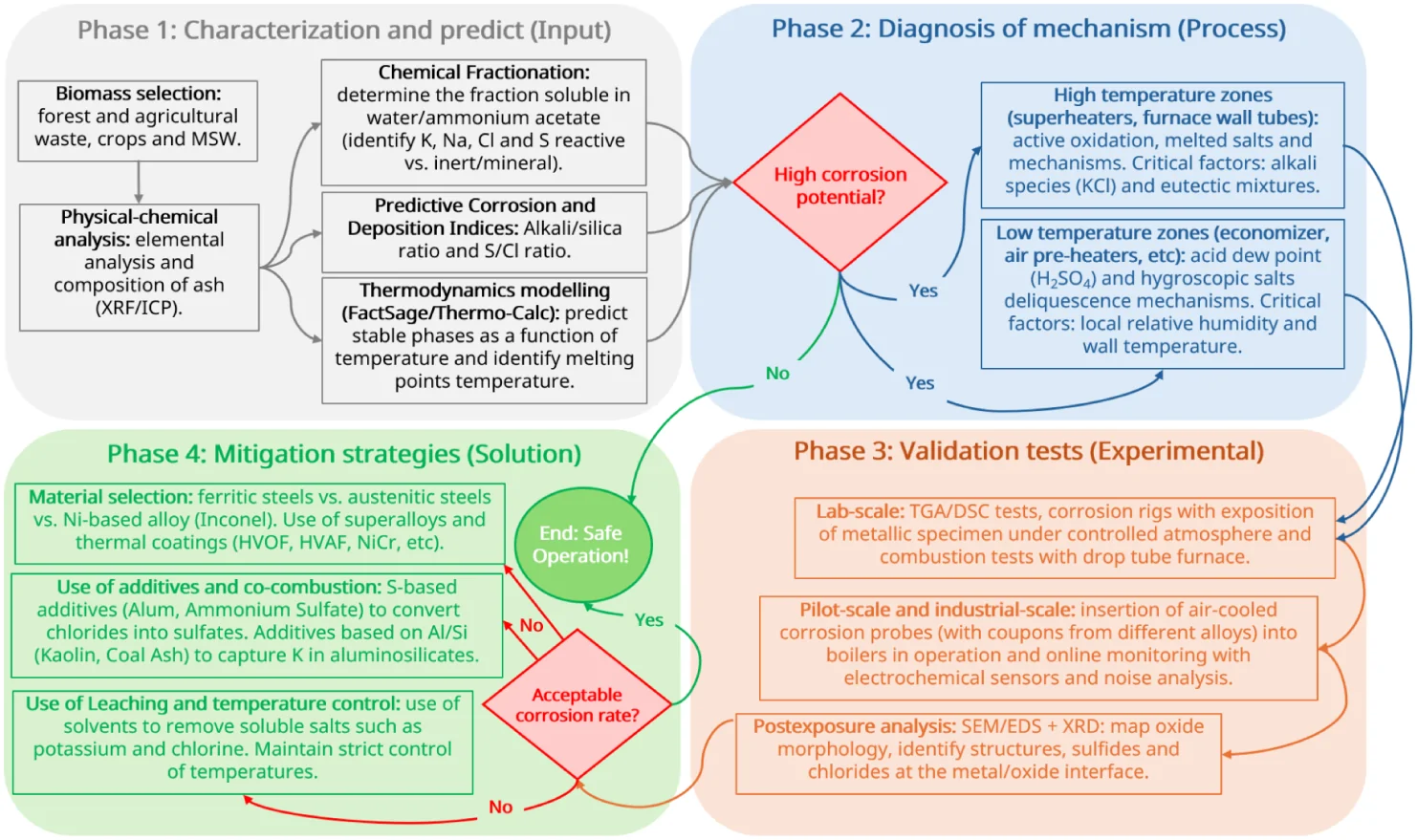
Investigative methodology of deposition and corrosion problems in boilers.

**Table 7:** Review of Experimental Literature on High-Temperature Corrosion in Biomass Boilers

**Scope**	**Fuel**	**Scale**	**Temperature**	**Methods**	**Objective**	**ref.**
Corrosion resistance of 347H steel with and without NiCr coating	Rice Husk	Industrial	700–800 °C	MEV-EDS and XRD characterization, plus weight loss of probes	Characterization/Mitigation	^162^
Characterization and mathematics adjust for corrosion products by ash deposition	Straw, corn stalk, cotton and coal blends	Laboratory	600 °C	Gravimetric technics (static weight gain of specimens)	Characterization/Quantification	^22^
Corrosion resistance of thermal coatings (HVOF, PTA) via melted ashes	MSW^a^	Laboratory	450–650 °C	MEV, XRD characterization and corrosion/adhesion tests	Mitigation	^163^
Time and temperature effects on morphology of deposits by oxidative gases exposition	Synthetic salts	Laboratory	500–800 °C	MEV-EDS and XRD characterization, plus thermodynamics modeling	Characterization/Mechanistic approach	^164^
Hot corrosion analysis for amorphous coating Fe-based (Fe–Cr–Mo)	Biomass^a^	Laboratory	500–600 °C	MEV-EDS, DRX, ICP-OES characterization and 3D profile	Mechanistic approach/Mitigation	^165^
Gaseous K capture by H_2_SO_4_ via KCl sulphation	Biomass^a^ and MSW	Laboratory	600–1250 °C	Elemental analysis, gas measuring and kinetic/thermodynamic modeling via Chemkim-Pro	Quantification/Mechanistic approach	^27^
Influence of the addition of fly ash on slagging and deposition	Rice Husk	Pilot (30 kW)	1000–1200 °C	MEV-EDS, DRX and XRF characterization, plus microscopic optic	Characterization/Mitigation	^166^
Effects of additives CFA and kaolin on aerosols and deposition formation	Wood pellets, wheat straw	Laboratory/Pilot/Industrial (800–900 MW)	550–933 °C	MEV-EDS and DRX characterization, plus deposition probes utilization and impactor (ELPI)	Characterization/Mitigation	^100^
Corrosion resistance analyzed by influence of surface conditions (ash/salts) and Inconel 625 coating	MSW	Laboratory/Industrial	380–600 °C	MEV-EDS and DRX characterization, plus kinetic analysis by mass gain technic	Characterization/Quantification/Mitigation	^167^
Temperature and humidity effects on corrosion of 12Cr1MoVG steel	MSW	Laboratory	600 °C	MEV-EDS and DRX characterization, plus kinetic analysis by mass gain technic	Characterization/Quantification/Mechanistic approach	^168^
Effectiveness of coatings (Inconel 625/686) for corrosion resistance under chlorine rich ash deposition	MSW^a^ and biomass	Laboratory	480–580 °C	MEV-EDS characterization and gravimetric analysis	Quantification/Mitigation	^169^
Effectiveness of dry torrefaction process on chlorine concentration reduction of *Eucalyptus* sp. for combustion	*Eucalyptus* sp.	Laboratory	260 °C	Dry torrefaction thermal processing, chlorine determination and statistical analysis	Mitigation	^170^
Potassium release during combustion on different boilers configuration (grate, fixed bed and suspension) and mitigation strategies	Wood, straw, miscanthus, etc	Laboratory/Pilot/Industrial	600–1700 °C	ETV-ICP-OES technics and thermodynamic modeling via FactSage	Characterization/Quantification/Mitigation	^88^
Anti chlorine alkali salts attack performance of YSZ/NiCo coatings	MSW	Laboratory	600 °C	MEV-EDS characterization, electrochemical analysis and weight loss technic	Characterization/Mitigation	^171^
Anti corrosion/slagging performance of HVOF coatings (Inconel 625/718)	Wood chips, rice husk, forest crops	Industrial	800–900 °C	MEV-EDS and DRX characterization, plus oxide scale thickness measurement	Characterization/Mitigation	^172^
Anti corrosion performance of different boilers steels (T91, SS304, SS316, SS410)	Wood chips, rice husk, forest crops	Industrial (34,5 MW)	800–900 °C	MEV-EDS and DRX characterization, plus gravimetric technic	Characterization/Quantification/Mechanistic approach	^173^

a Simulated environments from analogous real conditions from respective heterogeneous fuels.

Table 7 illustrates a shift from reactive observation to predictive and systemic corrosion management. Findings indicate that effective mitigation requires integrating advanced metallurgy, chemical additives, and rigorous fuel pretreatment. However, current reliance on instrumental characterization, gravimetric kinetics, and thermal modeling limits structural failure predictability on industrial scales. Consequently, future research must prioritize coupled (gas-ash-metal) analyses under thermal gradients and long-term pilot-scale validation of laboratory findings.

## 8 The Brazilian Scene: A Consolidated Core and an Undocumented Frontier

Brazil began 2026 with approximately 216,000 MW of installed centralized generation capacity, 84.63% of it is renewable, and the biomass segment alone accounts for 18,130 MW distributed across 653 plants, having added 732 MW during 2025 and holding a further 1,827.5 MW licensed across 42 plants either under construction or awaiting it, roughly 10% of the existing fleet.^174,175^ This capacity is, however, far from homogeneous. Sugar cane bagasse supplies 12,356 MW across 387 plants, or 60.4% of national cogeneration, followed by black liquor with 3,407 MW across only 21 plants (16.6%).^176^ Solid bioenergy delivered 5.1% of the country’s electricity in 2024, with bagasse alone generating 37.4 TWh, with 16.0 TWh consumed on-site and 21.4 TWh exported to the grid.^177^

Approximately three-quarters of the Brazilian biomass-fired fleet therefore burns two fuels whose ash chemistry is comparatively well characterized and whose boilers have accumulated decades of operational experience. The corrosion problem addressed in this review is not primarily located there. It is located at the margins, and the margins is where the fleet is growing. Rice husk supports 14 dedicated thermal plants nationally, an order of magnitude below the bagasse fleet, yet these units operate at capacity factors of 85–90% with a calorific value comparable to that of mineral coal.^174^ This is precisely the attribute that bagasse cannot offer: cane cogeneration is bound to the harvest and delivers its surplus seasonally, whereas rice husk, corn cobs, wood chips, and short-rotation eucalyptus are available year-round and are dispatchable against the hydrological cycle. The agro-industrial residues that resolve the intermittency of the consolidated fleet are, however, the same ones that carry the adverse ash chemistry described in Sections 4 and 5: amorphous silica with high potassium in rice husk, potassium chloride in corn cobs, and, in eucalyptus, chlorine introduced through potassium-based fertilization during cultivation. The strategic value of these fuels and their corrosive severity have the same origin, and the boilers now being licensed will burn them using designs qualified for bagasse. Table 8 summarizes the current status of large-scale industrial stream boiler or other thermal biomass processing units in Brazil.

**Table 8:** Status of Biomass Stream Boilers in Brazil. Ref. [ ^174–177^ ]

**Feedstock**	**Installed capacity (MW)**	**No. of plants**	**Share of cogeneration**	**Capacity factor**
Sugar cane bagasse	12,356	387	60.4%	NA
Black liquor	3,407	21	16.6%	NA
Rice husk	No information	14 (dedicated)	NA	85–90%
**Total segment**	18,130	653	100%	NA
Under construction	1,827.5	42	(+10% of fleet)	NA

Barriers to corrosion mitigation persist across academia and industry because of economic constraints. Although aluminosilicate and phosphate additives are highly effective, their industrial scale cost is often prohibitive.

Any assessment of how severe this problem already is in Brazil confronts an absence, rather than a number. There is no public census of the industrial boiler fleet: NR-13 ^178^ mandates periodic inspection and the issuance of formal inspection reports for every boiler in the country, but these records are never aggregated and no national database consolidates them. There is, in consequence, no published corrosion-related tube failure rate, no mean time between superheater retubing, and no survey of the adoption rate of mitigation technologies, additive dosing, coating, cladding, or alloy upgrading across the fleet. Brazil has no equivalent to the National Board of Boiler and Pressure Vessel Inspectors, which has maintained standardized incident reporting in the United States since 1991. The only quantified series available derives from the technical justification for the revision of NR-13, which recorded approximately 70 events involving boilers, pressure vessels, and piping between 2003 and 2012, of which 22 involved boilers, causing 20 fatalities and 51 injuries.^178^ These are catastrophic failures, not degradation statistics: they record explosions, not the tube-wall thinning that determines when a superheater is replaced. The distinction matters because it is the second category that governs the economics of biomass firing and the first that governs regulation.

This gap is not exclusive to Brazil. Harun and Islam^179^ identify the scarcity of standardized, openly available corrosion data sets as the principal obstacle to reproducible model benchmarking worldwide, with the ISO–CORRAG atmospheric database as a rare exception and no equivalent whatsoever for boiler exposures. What is specific to Brazil is the degree: the country lacks not only the exposure data set but the denominator—the number, age, fuel, and design of the boilers themselves. Any attempt to prioritize mitigation investment, to model the residual life of the fleet, or to train a data-driven predictive tool on national conditions is therefore blocked at the point of data collection rather than at the point of analysis. No sector-specific estimate exists for the cost of high-temperature corrosion in Brazilian biomass boilers. Figures in circulation derive from the Koch et al.^180^ study, which estimated the global cost of corrosion at US$ 2.5 trillion, or 3.4% of world GDP, in 2013, and which the Brazilian Corrosion Association applies proportionally to national GDP,^181^ while a competing extrapolation attributes 4% of GDP, or R$ 236 billion in 2015, to the same phenomenon. Both are pro-rated all-sector values rather than national measurements, and neither disaggregates by industry, which renders them unusable as evidence of a technological gap in biomass thermal conversion. The closest available analogue for the dominant Brazilian segment is international: Sugar cane Research Australia estimates approximately five million dollars annually for the Australian sugar milling industry in repair and downtime arising from inefficient boiler operation.^156^ No Brazilian equivalent has been published for any fuel.

Nevertheless, in SouthernBrazil industrial sludges from the metallurgical, textile, and pulp and paper sectors, possibly rich in Al, Ca, and P, offer a viable alternative. Santa Catarina offers a concrete opportunity to substitute high-purity additives with industrial residues. A diagnostic assessment of industrial solid waste in the state, based on the Waste Transport Manifests issued by the Santa Catarina Environmental Institute (IMA) for 2020–2021, found that thermal-process residues account for 25% of the industrial waste stream and pulp-and-paper residues for a further 35%, with generation concentrated in the Joinville, Criciúma, and Blumenau regional districts; of the total, 45.5% is still landfilled and only 35.7% is recycled.^182^ The scale and low valorization of these streams, together with their proximity to the biomass-firing industry of the region, make them candidate raw materials for antislagging additive formulation. Metallurgical, textile, and pulp-and-paper sludges are reported to be enriched in aluminum, calcium, and phosphorus, the same elements that raise ash-fusion temperatures through the mechanisms described in Section 6.2, which would, in principle, allow them to act as alkali scavengers in place of high-purity kaolin or phosphate additives, reducing both additive cost and the environmental burden of landfilling. However, this possibility remains to be verified: the oxide composition, reactivity, and ash-fusion behavior of these specific residues have not been characterized, and their qualification as biomass additives is identified here as a research direction rather than an established practice.

The demand for viable mitigation is critical in Brazil, where the industrial energy matrix relies heavily on rice-husk and wood-chip combustion. Practical constraints, such as continuous steam requirements and high costs, often render leaching and the usen of superalloys to be unfeasible for traditional sectors. Addressing these limitations is a prerequisite for the inevitable transition to municipal solid waste (MSW) energy recovery,^183^ a model already established in leaders like Denmark and China. However, MSW introduces severe corrosion risks because of high chlorine and heavy metal loads. Consequently, Brazil must foster its national metallurgical and boiler manufacturing industries, evolving from conventional equipment to specialized alloys and designs tailored to complex combustion environments. Without technological progress in materials engineering, the country will remain dependent on foreign technology and lose its competitive advantage in the fuel volume.

## 9 Conclusions

Ash deposition and chlorine-driven active oxidation are mechanistically well established; what remains contested is the response. Mitigation is best framed as a single decision problem in which no option is simultaneously effective, inexpensive, and mature, and the correct choice depends on whether the fuel can be altered, the metal temperature, and the deposit chemistry.

Mitigation through cocombustion, leaching, and additives focuses on increasing ash fusion temperatures via alkali sequestration. Advanced metallic coatings offer superior protection but are limited by high implementation costs. Coating performance is governed by the continuity of the protective scale, not by nominal composition or porosity: denser cold-sprayed NiCr underperforms HVOF because its Cr_2_O_3_ layer is discontinuous. In the field, iron-based FeCrAl matched the nickel-based reference under alkali-chloride deposits, showing that the cost barrier to coatings lies in the nickel feedstock and not the process. Laboratory rate constants overestimate service life, since they omit the particle impact and soot blowing that make erosion, not corrosion, the discriminating variable in service.

For plants that cannot alter their fuel, additive dosing and selective coating of the hottest passes are complementary levers. Iron-based FeCrAl and FeCr should be evaluated before nickel superalloys but qualified under actual chloride deposits rather than assumed protective; aluminide coatings should not be specified for chloride service without prior oxide conditioning; and antislagging measures must be weighed against the metal loss that soot-blowing accelerates.

No standard quantifies antislagging capability or clad thickness on tubes; no open exposure data set exists, so AI prediction remains unvalidated in the molten-alkali regime and is weakest at high corrosion rates, while the chemistry of residue-derived additives is uncharacterized. Five priorities follow: open Brazilian ash and exposure databases seeded from NR-13 inspection records; CFD–corrosion coupling extending existing bagasse flow-field models; physics-informed rather than black-box AI prediction; coatings qualified under KCl–K_2_SO_4_–SiO_2_ deposits in operating boilers; and long-term field studies able to resolve slow processes such as 475 °C embrittlement.

Thus, Brazil possesses abundant biomass resources but requires further R&D for full exploitation. Utilizing local industrial byproducts as corrosion inhibitors offers a sustainable and economically viable path. Future strategies must prioritize Municipal Solid Waste (MSW) energy recovery to align with global technological standards. Roughly three-quarters of installed biomass capacity burns bagasse and black liquor, yet the transition’s strategic value lies in rice husk, corn cobs, and eucalyptus, dispatchable year-round but far more corrosive. Brazil is thus preparing to burn its most corrosive fuels in equipment qualified for the least corrosive ones, without the data to manage the risk: no fleet census, no failure statistics, only a prorated global cost figure. The binding constraint is not a shortage of mitigation technology but of data and domestic materials-engineering capacity. Two national assets could change this: (i) the inspection records that NR-13 already mandates and (ii) the underused industrial residues of states such as Santa Catarina—and the coming shift to municipal-solid-waste recovery makes acting on them urgent. Ceramic coatings and AI-based predictive monitoring (machine learning, digital twins) remain comparatively underexplored in the biomass-boiler corrosion literature and are flagged here as priority directions for future review and experimental work.

## References

[1] Zhang Y. X., Luo H. L., Wang C. (2021). Progress and trends of global carbon
neutrality pledges. Clim. Change Res.

[2] Nguyen H. H. T., Nguyen H. T., Ahmed S. F., Rajamohan N., Yusuf M., Sharma A., Arunkumar P., Deepanraj B., Tran H.-T., Al-Gheethi A., Vo D.-V. N. (2023). Emerging waste-to-wealth applications of fly ash for
environmental remediation: A review. Environ.
Res.

[3] Yusuf M., Ibrahim H. (2023). A comprehensive review
on recent trends in carbon capture,
utilization, and storage techniques. J. Environ.
Chem. Eng.

[4] Liu L., Memon M. Z., Xie Y., Gao S., Guo Y., Dong J. (2023). Recent advances of
research in coal and biomass co-firing for electricity
and heat generation. Circular Economy.

[5] Le
Quéré C., Jackson R. B., Jones M. W., Smith A. J. P., Abernethy S., Andrew R. M., De-Gol A. J., Willis D. R., Shan Y., Canadell J. G. (2020). Temporary reduction
in daily global CO2 emissions during the COVID-19 forced confinement. Nat. Clim. Change.

[6] Companhia Nacional de Abastecimento (CONAB). Produção de grãos e área plantada da safra 2025/26 mantém perspectiva de novos recordes; CONAB, Brasília, 2026. https://www.noticiasagricolas.com.br/noticias/graos/413957-producao-de-graos-e-area-plantada-da-safra-2025-26-mantem-perspectiva-de-novos-recordes.html (Accessed 10 April 2026).

[7] Balan V. (2014). Current challenges
in commercially producing biofuels from lignocellulosic biomass. ISRN Biotechnol.

[8] Saidur R., Abdelaziz E. A., Demirbas A., Hossain M. S., Mekhilef S. (2011). A review on
biomass as a fuel for boilers. Renewable Sustainable
Energy Rev.

[9] Beims R. F., Simonato C. L., Wiggers V. R. (2019). Technology readiness
level assessment
of pyrolysis of triglyceride biomass to fuels and chemicals. Renewable Sustainable Energy Rev.

[10] Basu P., Kaushal P. (2009). Modeling of pyrolysis
and gasification of biomass in
fluidized beds: A review. Chem. Prod. Process
Model.

[11] Bridgwater A. V. (2012). Review
of fast pyrolysis of biomass and product upgrading. Biomass Bioenergy.

[12] Duffy N. T. M., Eaton J. A. (2013). Investigation of
factors affecting channelling in fixed-bed
solid fuel combustion using CFD. Combust. Flame.

[13] Mola-Yudego B., Dimitriou I., Gagnon B., Schweinle J., Kulišić B. (2024). Priorities
for the sustainability
criteria of biomass supply chains for energy. J. Clean. Prod.

[14] Cha S. C. (2007). High temperature
corrosion of superheater materials below deposited biomass ashes in
biomass combusting atmospheres. Corros. Eng.,
Sci. Technol.

[15] Abioye K. J., Harun N. Y., Sufian S., Yusuf M., Jagaba A. H., Ekeoma B. C., Kamyab H., Sikiru S., Waqas S., Ibrahim H. (2024). A review of biomass
ash related problems: Mechanism,
solution, and outlook. J. Energy Inst.

[16] Harding N. S., O’connor D. C. (2007). Ash deposition impacts in the power
industry. Fuel Process. Technol.

[17] Cerqueira D. P., Portella K. F., Portella G. D. O. G., Cabussú M., Machado E. C., da Silva G. C., Brambilla K. J. C., de Oliveira Júnior D. R., Salles R. N., Pereira P. A. M. (2012). Deterioration rates of metal and concrete structures
in coastal environment of the South and Northeast Brazil: case studies
in the Pontal do Sul, PR, and Costa do Sauípe, Bahia. Procedia Eng.

[18] Sharma S., Sharma M., Mudgal D., Bhowmick H. (2021). Adoption of strategies
for clean combustion of biomass in boilers. Corros. Rev.

[19] Lee, S. H. ; Themelis, N. I. ; Castaldi, M. J. Combating corrosion in WTE facilities – Theory and experience. In Proceedings of the 14th Annual North American Waste-to-Energy Conference, ASME, Tampa, Florida, USA, 2006; pp 1–10. DOI: 10.1115/NAWTEC14-3198.

[20] Kumar S., Handa A., Chawla V., Grover N. K., Kumar R. (2021). Performance
of thermal-sprayed coatings to combat hot corrosion of coal-fired
boiler tube and effect of process parameters and post-coating heat
treatment on coating performance: a review. Surf. Eng.

[21] Blomberg T. (2011). A thermodynamic
study of the gaseous potassium chemistry in the convection sections
of biomass fired boilers. Mater. Corros.

[22] Wang Y., Sun Y., Yue M., Li Y. (2020). Reaction kinetics of chlorine corrosion
to heating surfaces during coal and biomass cofiring. J. Chem.

[23] Kaur G., Sharma N. K., Kaur J., Bajaj M., Zawbaa H. M., Turky R. A., Kamel S. (2022). Prospects
of biogas and evaluation
of unseen livestock based resource potential as distributed generation
in India. Ain Shams Eng. J.

[24] Berlanga C., Ruiz J. A. (2013). Study of corrosion
in a biomass boiler. J. Chem.

[25] Cao W., Li J., Zhang X. (2022). Evaluation of the effects and interactions of initial
chlorine and sulphur contents on the release of potassium compounds
during biomass combustion. J. Energy Inst.

[26] IEA Bioenergy. Implementation of bioenergy in Brazil - 2024 update; IEA Bioenergy Technology Collaboration Programme, 2024.

[27] Chanpirak A., Jensen M. K. K., Wu H., Glarborg P. (2023). Sulfation of gaseous
KCl by H2SO4. Energy Fuels.

[28] Antunes R. A., de Oliveira M. C. L. (2013). Corrosion
in biomass combustion: A materials selection
analysis and its interaction with corrosion mechanisms and mitigation
strategies. Corros. Sci.

[29] Niu Y., Tan H., Hui S. (2016). Ash-related
issues during biomass combustion: Alkali-induced
slagging, silicate melt-induced slagging (ash fusion), agglomeration,
corrosion, ash utilization, and related countermeasures. Prog. Energy Combust. Sci.

[30] Hu S., Finkle H., Liu X. (2021). A review on molten sulfate salts
induced hot corrosion. J. Mater. Sci. Technol.

[31] Li K., Zeng Y. (2022). Corrosion of heat exchanger
materials in co-combustion thermal power
plants. Renewable Sustainable Energy Rev.

[32] Maj I., Niesporek K., Płaza P., Maier J., Łój P. (2025). Biomass ash:
A review of chemical compositions and management trends. Sustainability.

[33] Page M. J., McKenzie J. E., Bossuyt P. M., Boutron I., Hoffmann T. C., Mulrow C. D., Shamseer L., Tetzlaff J. M., Akl E. A., Brennan S. E. (2021). The PRISMA 2020 Statement: An Updated Guideline
for Reporting Systematic Reviews. BMJ.

[34] Guo X., Wang S., Wang Q., Guo Z., Luo Z. (2011). Properties
of bio-oil from fast pyrolysis of rice husk. Chin. J. Chem. Eng.

[35] Ang T. N., Ngoh G. C., Chua A. S. M., Lee M. G. (2012). Elucidation
of the
effect of ionic liquid pretreatment on rice husk via structural analyses. Biotechnol. Biofuels.

[36] Abu
Bakar M. S., Titiloye J. O. (2013). Catalytic pyrolysis of rice husk
for bio-oil production. J. Anal. Appl. Pyrolysis.

[37] Lopes E. R., Medina A. L., Ribeiro A. S., Brandalise J. N., Nunes A. M. (2017). Elementary characterization of rice
husk and their
ashes by MIP OES after acid decomposition with reflux system. Quim. Nova.

[38] Carvalho A., Rabaçal M., Costa M., Alzueta M. U., Abián M. (2017). Effects of
potassium and calcium on the early stages of combustion of single
biomass particles. Fuel.

[39] Cao W., Li J., Martí-Rosselló T., Zhang X. (2019). Experimental study
on the ignition characteristics of cellulose, hemicellulose, lignin
and their mixtures. J. Energy Inst.

[40] Xue Q., Heindel T. J., Fox R. O. (2011). A CFD model
for biomass fast pyrolysis
in fluidized-bed reactors. Chem. Eng. Sci.

[41] Vassilev S. V., Baxter D., Andersen L. K., Vassileva C. G., Morgan T. J. (2012). An overview of the organic and inorganic
phase composition
of biomass. Fuel.

[42] Blomberg T., Makkonen P., Hiltunen M. (2004). Role of alkali hydroxides
in the
fireside corrosion of heat transfer surfaces, a practical approach. Mater. Sci. Forum.

[43] Brossard J. M., Diop I., Chaucherie X., Nicol F., Rapin C., Vilasi M. (2011). Superheater fireside
corrosion mechanisms in MSWI plants:
Lab-scale study and on-site results. Mater.
Corros.

[44] Awulu J. O., Omale P. A., Ameh J. A. (2018). Comparative analysis of calorific
values of selected agricultural wastes. Niger.
J. Technol.

[45] Armynah B., Piarah Z., Djafar W. H., Tahir D., Tahir D. (2018). Analysis of
chemical and physical properties of biochar from rice husk biomass. J. Phys.: conf. Ser.

[46] Bryers R. W. (1996). Fireside
slagging, fouling, and high-temperature corrosion of heat-transfer
surface due to impurities in steam-raising fuels. Prog. Energy Combust. Sci.

[47] Vamvuka D., Zografos D. (2004). Predicting the behaviour
of ash from agricultural wastes
during combustion. Fuel.

[48] Demirbas A. (2007). Combustion
of biomass. Energy Sources, Part A.

[49] Tortosa
Masiá A. A., Buhre B. J. P., Gupta R. P., Wall T. F. (2007). Characterising
ash of biomass and waste. Fuel Process. Technol.

[50] Lapuerta M., Hernández J. J., Rodríguez J. (2004). Kinetics of
devolatilisation of forestry
wastes from thermogravimetric analysis. Biomass
Bioenergy.

[51] Lewandowski I., Clifton-Brown J. C., Scurlock J. M. O., Huisman W. (2000). Miscanthus: European
experience with a novel energy crop. Biomass
Bioenergy.

[52] Moilanen, A. Thermogravimetric characterisations of biomass and waste for gasification processes; VTT Publications: Espoo, 2006.

[53] Madhiyanon T., Sathitruangsak P., Sungworagarn S., Udomman T. (2020). Investigation of rice-straw-ash
fouling/slagging and countermeasures using supplementary additives
and co-firing with Si-Al-rich coal in a pilot-scale grate-fired combustor. J. Energy Inst.

[54] Fernandes I. J., Calheiro D., Kieling A. G., Moraes C. A. M., Rocha T. L. A. C., Brehm F. A., Modolo R. C. E. (2016). Characterization
of rice husk ash
produced using different biomass combustion techniques for energy. Fuel.

[55] Albuquerque A. R. L., Angélica R. S., Merino A., Paz S. P. A. (2021). Chemical
and mineralogical characterization and potential use of ash from Amazonian
biomasses as an agricultural fertilizer and for soil amendment. J. Clean. Prod.

[56] de
Palma K. R., Tomaz E., Soria-Verdugo A., Silva M. A. (2021). The influence of the elemental and structural chemical
composition on the ash fusibility of sugarcane bagasse and sugarcane
straw. Fuel.

[57] Lariot-Sánchez C., Rivas-Gutierrez A., Rodríguez-Machín L., Rubio-González A., Iturria Quintero P. J. (2022). Impact
of alkalis and chlorides from sugarcane agriculture residues on high
temperature corrosion: a review. Oxid. Met.

[58] Filho P. A., Badr O. (2004). Biomass resources for
energy in North-Eastern Brazil. Appl. Energy.

[59] Lora E. S., Andrade R. V. (2009). Biomass as energy
source in Brazil. Renewable Sustainable Energy
Rev.

[60] Ferreira-Leitão V., Gottschalk L. M. F., Ferrara M. A., Nepomuceno A. L., Molinari H. B. C., Bon E. P. S. (2010). Biomass
residues in Brazil: availability
and potential uses. Waste Biomass Valor.

[61] Lima M. A., Gomez L. D., Steele-King C. G., Simister R., Bernardinelli O. D., Carvalho M. A., Rezende C. A., Labate C. A., Azevedo E. R., McQueen-Mason S. J. (2014). Evaluating the composition and processing
potential of novel sources of Brazilian biomass for sustainable biorenewables
production. Biotechnol. Biofuels.

[62] Rocha E. P. A., Gomes F. J. B., Sermyagina E., Cardoso M., Colodette J. L. (2015). Analysis
of Brazilian biomass focusing on thermochemical conversion for energy
production. Energy Fuels.

[63] de
Luna M. Y., Rodrigues P. M., Gadelha A. M. T., da
Costa Júnior A. E., Malveira J. Q., Mazzetto S. E., Sousa R. M. A. (2019). A thermogravimetric analysis of biomass wastes from
the northeast region of Brazil as fuels for energy recovery. Energy Sources, Part A.

[64] da
Silva C. M. S., Carneiro A. C. O., Vital B. R., Figueiró C. G., Fialho L. F., Magalhães M. A., Carvalho A., Cândido G. W. L. (2018). Biomass
torrefaction for energy purposes – Definitions and an overview
of challenges and opportunities in Brazil. Renewable
Sustainable Energy Rev.

[65] Hansted A. L. S., Cacuro T. A., Nakashima G. T., Costa V. E., Yamamoto H., Yamaji F. M. (2018). Use of a lignocellulosic residue as solid fuel: The
effect of ash content in the energy potential. Ind. Crops Prod.

[66] da
Silva S. B., Arantes M. D. C., de Andrade J. K. B., Andrade C. R., Carneiro A. C. O., Protásio T. P. (2020). Influence
of physical and chemical compositions on the properties and energy
use of lignocellulosic biomass pellets in Brazil. Renewable Energy.

[67] Wiggers V. R., Wisniewski A., Madureira L. A. S., Barros A. A. C., Meier H. F. (2009). Biofuels
from waste fish oil pyrolysis: continuous production in a pilot plant. Fuel.

[68] Wiggers V. R., Meier H. F., Wisniewski A., Barros A. A. C., Maciel M. R. W. (2009). Biofuels
from continuous fast pyrolysis of soybean oil: A pilot plant study. Bioresour. Technol.

[69] Ben
Atitallah I., Antonopoulou G., Ntaikou I., Alexandropoulou M., Nasri M., Mechichi T., Lyberatos G. (2019). On the evaluation
of different saccharification schemes for enhanced bioethanol production
from potato peels waste via a newly isolated yeast strain of Wickerhamomyces
anomalus. Bioresour. Technol.

[70] Xia C., Cai L., Zhang H., Zuo L., Shi S. Q., Lam S. S. (2021). A review
on the modeling and validation of biomass pyrolysis with a focus on
product yield and composition. Biofuel Res.
J.

[71] Dias D., Lapa N., Bernardo M., Godinho D., Fonseca I., Miranda M., Pinto F., Lemos F. (2017). Properties of chars
from the gasification and pyrolysis of rice waste streams towards
their valorisation as adsorbent materials. Waste
Manage.

[72] Maga D., Thonemann N., Hiebel M., Sebastião D., Lopes T. F., Fonseca C., Gírio F. (2019). Comparative
life cycle assessment of first- and second-generation ethanol from
sugarcane in Brazil. Int. J. Life Cycle Assess.

[73] Kwon G., Bhatnagar A., Wang H., Kwon E. E., Song H. (2020). A review of
recent advancements in utilization of biomass and industrial wastes
into engineered biochar. J. Hazard. Mater.

[74] Lora, E. ; Andrade, R. V. ; Angel, J. D. M. ; Leite, M. A. H. ; Rocha, M. H. ; de Sales, C. A. V. B. ; Mendoza, M. A. G. ; Coral, D. S. O. Gaseificação e pirólise para a conversão da biomassa em eletricidade e biocombustíveis. In Biocombustíveis, Interciência, 2012.

[75] Yin C., Li S. (2017). Advancing grate-firing
for greater environmental impacts and efficiency
for decentralized biomass/wastes combustion. Energy Procedia.

[76] Egsgaard, H. ; Hansen, U. ; Jensen, P. A. ; Glarborg, P. ; Nielsen, C. Combustion and gasification technologies. In Risø energy report 2: New and emerging bioenergy technologies; Larsen, H. ; Kossmann, J. ; Petersen, L. S. , Eds.; Risø National Laboratory: Roskilde, 2003; pp. 35–39.

[77] Brand M. A., Henne R. A., Schein V. A. S., Pereira E. R. (2021). Problem mapping
of the generation and treatment of forest biomass ashes in boiler. Cienc. Florest.

[78] Kumar A., Kumar N., Baredar P., Shukla A. (2015). A review on biomass
energy resources, potential, conversion and policy in India. Renewable Sustainable Energy Rev.

[79] Yildiz C., Richter M., Ströhle J., Epple B. (2023). Release of sulfur and
chlorine gas species during combustion and pyrolysis of walnut shells
in an entrained flow reactor. Energies.

[80] Trubetskaya A., Jensen P. A., Jensen A. D., Garcia Llamas A. D., Umeki K., Glarborg P. (2016). Effect of fast pyrolysis conditions
on biomass solid residues at high temperatures. Fuel Process. Technol.

[81] Hindiyarti L., Frandsen F., Livbjerg H., Glarborg P., Marshall P. (2008). An exploratory
study of alkali sulfate aerosol formation during biomass combustion. Fuel.

[82] van Loo, S. ; Koppejan, J. Biomass ash characteristics and behaviour in combustion systems; IEA Bioenergy, 2013.

[83] Wang X., Hu Z., Adeosun A., Liu B., Ruan R., Li S., Tan H. (2018). Particulate matter emission and K/S/Cl transformation during biomass
combustion in an entrained flow reactor. J.
Energy Inst.

[84] Bashir M. S., Jensen P. A., Frandsen F., Wedel S., Dam-Johansen K., Wadenbäck J., Pedersen S. T. (2012). Ash transformation and deposit build-up
during biomass suspension and grate firing: Full-scale experimental
studies. Fuel Process. Technol.

[85] Krause H. H. (1986). High temperature
corrosion problems in waste incineration systems. J. Mater. Energy Syst.

[86] Baxter L. L., Miles T. R., Miles T. R., Jenkins B. M., Milne T., Dayton D., Bryers R. W., Oden L. L. (1998). The behavior
of inorganic material in biomass-fired power boilers: field and laboratory
experiences. Fuel Process. Technol.

[87] Chen C., Luo Z., Yu C., Wang T., Zhang H. (2017). Transformation behavior
of potassium during pyrolysis of biomass. RSC
Adv.

[88] Mörtenkötter H., Kerscher F., Schönsteiner M., DeYoung S., Fendt S., Spliethoff H. (2024). Potassium release and mitigation by additives in different
biomass combustion systems. Fuel.

[89] Chen C., Bi Y., Huang Y., Huang H. (2021). Review on slagging evaluation methods
of biomass fuel combustion. J. Anal. Appl. Pyrolysis.

[90] Kleinhans U., Wieland C., Frandsen F. J., Spliethoff H. (2018). Ash formation
and deposition in coal and biomass fired combustion systems: Progress
and challenges in the field of ash particle sticking and rebound behavior. Prog. Energy Combust. Sci.

[91] Qiu T., Su H., Zhu Y., Zhai Y., Mikulčić H., Wang X., Xie J., Yang T. (2025). An integrated slagging
model of biomass-fired boiler combining condensation fouling and ash
viscous deposition. Renewable Energy.

[92] Sun Y., Sun J., Ma D., Qiu T., Zhu Y., Yang S., Wang X. (2026). Study on multiple mechanisms
and contribution weights of slagging
in biomass-fired boiler. Therm Sci.

[93] Wu D., Yuan Z., Liu S., Zheng J., Wei X., Zhang C. (2020). Recent development
of corrosion factors and coating applications
in biomass firing plants. Coatings.

[94] Vainio E., Laurén T., De Martini N., Brink A., Hupa M. (2014). Understanding
low-temperature corrosion in recovery boilers: risk of sulphuric acid
dew point corrosion?. J-FOR.

[95] Vainio E., Yrjas P., Hupa L., Hupa M. (2023). Cold-end corrosion
caused by hygroscopic ammonium chloride in thermal conversion of biomass
and waste. Fuel.

[96] Ruozzi A., Vainio E., Kinnunen H., Hupa L. (2023). Cold-end corrosion
in biomass combustion - Role of calcium chloride in the deposit. Fuel.

[97] Jafarihonar F., Vainio E., Hupa L., Hupa M. (2025). Low-temperature corrosion
in large-scale biomass boilers. NPJ. Mater.
Degrad.

[98] Montgomery M., Vilhelmsen T., Jensen S. A. (2008). Potential high temperature corrosion
problems due to co-firing of biomass and fossil fuels. Mater. Corros.

[99] Okoro S. C., Montgomery M., Frandsen F. J., Pantleon K. (2018). Time and temperature
effects on alkali chloride induced high temperature corrosion of superheaters
during biomass firing. Energy Fuels.

[100] Frandsen F. J., Jensen P. A., Wang G., Delgado R. N., de Riese T., Fendt S., Sander B. (2023). Ash deposition and
fine particle formation when utilizing biomass and coal fly ash in
lab-, pilot- and full-scale. Fuel.

[101] Tillman D. A., Duong D., Miller B. (2009). Chlorine in
solid fuels
fired in pulverized fuel boilers: Sources, forms, reactions, and consequences:
A literature review. Energy Fuels.

[102] Hong K., Zhou T., Zhang M., Zeng Y., Li W., Yang H. (2025). Combustion utilization
of high-chlorine coal: Current
status and future prospects. Energies.

[103] Obernberger I., Brunner T., Bärnthaler G. (2006). Chemical properties
of solid biofuels - Significance and impact. Biomass Bioenergy.

[104] Björkman E., Strömberg B. (1997). Release of
chlorine from biomass
at pyrolysis and gasification conditions. Energy
Fuels.

[105] Johansen J. M., Jakobsen J. G., Frandsen F. J., Glarborg P. (2011). Release of
K, Cl, and S during pyrolysis and combustion of high-chlorine biomass. Energy Fuels.

[106] Wu D., Li Y., Zheng J., Xiang L., Zhang C. (2025). Influence
of sulfur dioxide on the high temperature corrosion performance of
TP347H alloy and titanium aluminide coatings in simulated waste incineration
environments. Mater. Today Commun.

[107] Grabke H. J., Reese E., Spiegel M. (1995). The effects
of chlorides,
hydrogen chloride, and sulfur dioxide in the oxidation of steels below
deposits. Corros. Sci.

[108] Król D., Motyl P., Poskrobko S. (2022). Chlorine corrosion
in a low-power boiler fired with agricultural biomass. Energies.

[109] Wu S., Hu Y., Chen D., Wu N., Zhao X., Liu Z., Zhang R., Tang Y., Feng Y. (2024). Chlorine-induced gas-solid
coupled corrosion behaviors of tube materials in high steam parameter
MSW incinerator. Waste Manage.

[110] Santos D. (2013). Valorização energética
de resíduos
sólidos urbanos: materiais para caldeiras de centrais de incineração. Cienc. Tecnol. Mater.

[111] Mlonka-Mędrala A., Magdziarz A., Kalemba-Rec I., Nowak W. (2019). The influence of potassium-rich biomass
ashes on steel corrosion above 550 °C. Energy Convers. Manage.

[112] Sharp, W. B. A. Superheater corrosion in biomass boilers: today’s science and technology; Oak Ridge National Laboratory: Oak Ridge, 2011.

[113] Stringer J. (1977). Hot corrosion
of high-temperature alloys. Annu. Rev. Mater.
Sci.

[114] Eliaz N., Shemesh G., Latanision R. M. (2002). Hot corrosion
in gas turbine components. Eng. Failure Anal.

[115] Lehmusto J., Laurén T., Lindgren M. (2019). Catalytic Role of Process
Dust in SO2-to-SO3 Conversion in Flash Smelting Heat Recovery Boilers. JOM.

[116] Li H., Liu H., Huang Y., Chen X., Zhang X., Li J., Xu L., Yao H. (2024). Mechanism of coupling corrosion caused
by flue gas and deposits in municipal solid waste incinerator: Roles
of H2O­(g), HCl, and SO2. Corros. Sci.

[117] Mayoral M. C., Andrés J. M., Laguarta S. (2016). Evidence of corrosion
on metallic surfaces at 500 and 560 °C by metal-potassium trisulphate
formation in oxy-co-combustion. Fuel.

[118] Baker, B. A. ; Smith, G. D. Performance of Commercial Alloys in Simulated Waste Incineration Environments. In CORROSION 2001, NACE International, New Orleans, LA, USA, 2001. DOI: 10.5006/c2001-01183.

[119] Hussain T., Syed A. U., Simms N. J. (2013). Trends in fireside
corrosion damage to superheaters in air and oxy-firing of coal/biomass. Fuel.

[120] Huang, J. ; Lu, J. ; Yang, Z. ; Zhou, Y. ; Dang, Y. ; Yuan, Y. Corrosion behavior of austenitic stainless steels utilized for coal-fired boilers in simulated coal-ash/high sulfur environments. In 2018 International Conference on Power System Technology (POWERCON), IEEE, Guangzhou, China, 2018; pp 1–6. DOI: 10.1109/POWERCON.2018.8601684.

[121] Tanaka, Y. ; Komai, N. ; Suganuma, H. Coal ash corrosion properties of Ni-based alloy for advanced-USC boilers. In Advances in Materials Technology for Fossil Power Plants: Proceedings from the Sixth International Conference, Gandy, D. ; Shingledecker, J. ; Viswanathan, R. , Eds.; ASM International, Santa Fe, New Mexico, USA, 2011; pp 303–311. DOI: 10.31399/asm.cp.am-epri-2010p0303.

[122] Mahajan S., Chibber R. (2019). Hot corrosion studies of boiler steels
exposed to different molten salt mixtures at 950 °C. Eng. Failure Anal.

[123] Mudgal D., Singh S., Prakash S. (2014). Corrosion
problems
in incinerators and biomass-fuel-fired boilers. Int. J. Corros.

[124] Weng W., Brackmann C., Leffler T., Aldén M., Li Z. (2019). Ultraviolet absorption
cross sections of KOH and KCl for nonintrusive
species-specific quantitative detection in hot flue gases. Anal. Chem.

[125] Luan J., Wang Q., Shao D., Cui B., Han P., He Q. (2025). Research progress on influencing
factors and control
methods of slagging in biomass combustion. Front.
Energy Res.

[126] Pettersson J., Folkeson N., Johansson L. G., Svensson J. E. (2011). The effects of KCl, K2SO4 and K2CO3 on the high temperature
corrosion of a 304-type austenitic stainless steel. Oxid. Met.

[127] Lehmusto J., Yrjas P., Skrifvars B. J., Hupa M. (2012). High temperature corrosion of superheater steels by KCl and K2CO3
under dry and wet conditions. Fuel Process.
Technol.

[128] Lehmusto J., Skrifvars B. J., Yrjas P., Hupa M. (2013). Comparison
of potassium chloride and potassium carbonate with respect to their
tendency to cause high temperature corrosion of stainless 304L steel. Fuel Process. Technol.

[129] Lindmark H., Faust R., Tommy S., Johansson L. G., Knutsson P., Liske J. (2025). Corrosivity of KOH­(g) towards superheater
materials in a simulated air reactor environment for chemical looping
combustion of biomass. Fuel.

[130] Michelsen H. P., Frandsen F., Dam-Johansen K., Larsen O. H. (1998). Deposition and high
temperature corrosion in a 10 MW
straw fired boiler. Fuel Process. Technol.

[131] Hindiyarti L., Glarborg P., Marshall P. (2007). Reactions
of SO3 with
the O/H Radical Pool under Combustion Conditions. J. Phys. Chem. A.

[132] Okoro S. C., Montgomery M., Frandsen F. J., Pantleon K. (2014). High temperature
corrosion under laboratory conditions simulating biomass-firing: A
comprehensive characterization of corrosion products. Energy Fuels.

[133] Carvalho N. R., Barros J. L., Silva D. A., Nakashima G. T., Yamaji F. M. (2020). Caracterização física e química
da biomassa usada como combustível sólido em uma caldeira. Quim. Nova.

[134] Lind T., Kauppinen E. I., Hokkinen J., Jokiniemi J. K., Orjala M., Aurela M., Hillamo R. (2006). Effect of Chlorine
and Sulfur on Fine Particle Formation in Pilot-Scale CFBC of Biomass. Energy Fuels.

[135] Yin J., Wu Z. (2009). Corrosion Behavior of TP316L of Superheater in Biomass
Boiler with Simulated Atmosphere and Deposit. Chin. J. Chem. Eng.

[136] Holcomb G. R., McGhee B. F., Fry A. T., Simms N. J., Davis K., Shim H. S., Bullard S. J. (2013). Boiler corrosion
and monitoring. Mater. High Temp.

[137] Li L., Ren Q., Li S., Lu Q. (2013). Effect of phosphorus
on the behavior of potassium during the co-combustion of wheat straw
with municipal sewage sludge. Energy Fuels.

[138] Matúš M., Križan P., Šooš Ĺ., Beniak J. (2018). The effect of papermaking
sludge as an additive to
biomass pellets on the final quality of the fuel. Fuel.

[139] Pan Z., Zhang S., Liu X., Zhang H., Zhu S. (2020). Effect of
Sludge-Based Additive on Ash Characteristic and Potassium Fixation
during the Rice Straw Combustion Process. Energy
Fuels.

[140] Wang T., Cai C., Xue Y., Xiao Y., Chen S., Liu J., Mei M., Li J. (2021). Regulation
of ash slagging behavior for sewage sludge by rice husk addition:
Focusing on control mechanisms. J. Clean. Prod.

[141] Karuana F., Prismantoko A., Suhendra N., Darmawan A., Hariana H., Darmadi D. B., Muflikhun M. A. (2023). Investigation
of austenitic stainless steel corrosion resistance against ash deposits
from co-combustion coal and biomass waste. Eng.
Failure Anal.

[142] Zhao Z., Yao X., Xu K., Li J. (2025). High-temperature
corrosion characteristics and mechanisms of heating surfaces under
ash deposition conditions in coal/biomass Co-firing utility boilers. J. Energy Inst.

[143] Liu H., Zhang L., Han Z., Xie B., Wu S. (2013). The effects
of leaching methods on the combustion characteristics of rice straw. Biomass Bioenergy.

[144] Carrillo M. A., Staggenborg S. A., Pineda J. A. (2014). Washing sorghum
biomass with water to improve its quality for combustion. Fuel.

[145] Werkelin J., Skrifvars B. J., Zevenhoven M., Holmbom B., Hupa M. (2010). Chemical forms
of ash-forming elements
in woody biomass fuels. Fuel.

[146] Saddawi A., Jones J. M., Williams A., Le Coeur C. (2012). Commodity
fuels from biomass through pretreatment and torrefaction: Effects
of mineral content on torrefied fuel characteristics and quality. Energy Fuels.

[147] Zevenhoven M., Yrjas P., Skrifvars B. J., Hupa M. (2012). Characterization of
ash-forming matter in various solid fuels by
selective leaching and its implications for fluidized-bed combustion. Energy Fuels.

[148] Gudka B., Jones J. M., Lea-Langton A. R., Williams A., Saddawi A. (2016). A review of the mitigation of deposition
and emission problems during biomass combustion through washing pre-treatment. J. Energy Inst.

[149] Yu C., Thy P., Wang L., Anderson S. N., Vander
Gheynst J. S., Upadhyaya S. K., Jenkins B. M. (2014). Influence of leaching
pretreatment on fuel properties of biomass. Fuel Process. Technol.

[150] Guo Q., Cheng Z., Chen G., Yan B., Hou L., Ronsse F. (2020). Optimal strategy for clean and efficient biomass combustion
based on ash deposition tendency and kinetic analysis. J. Clean. Prod.

[151] Míguez J. L., Porteiro J., Behrendt F., Blanco D., Patiño D., Dieguez-Alonso A. (2021). Review of the use of additives to
mitigate operational problems associated with the combustion of biomass
with high content in ash-forming species. Renewable
Sustainable Energy Rev.

[152] Abioye K. J., Harun N. Y., Sufian S., Yusuf M., Kamyab H., Hassan M. A., Jagaba A. H., Sikiru S., Ubaidullah M., Pandit B., Dhaliwal N. (2023). Regulation of ash slagging
behavior of palm oil decanter cake by alum sludge addition. Chemosphere.

[153] Wang L., Becidan M., Skreiberg Ø. (2012). Sintering
Behavior of Agricultural Residues Ashes and Effects of Additives. Energy Fuels.

[154] Wang L., Skreiberg Ø., Becidan M. (2014). Investigation of additives
for preventing ash fouling and sintering during barley straw combustion. Appl. Therm. Eng.

[155] Wang Q., Han K., Wang J., Gao J., Lu C. (2017). Influence of phosphorous based additives on ash melting
characteristics
during combustion of biomass briquette fuel. Renewable Energy.

[156] Khantisopon K., Singh S., Jitputti J., Berndt C. C., Ang A. S. M. (2024). High Temperature Corrosion Resistant and Anti-Slagging
Coatings for Boilers: A Review. High Temp. Corros.
Mater.

[157] Zhang X., Liu H., Chen T., Wang G., Li H., Hu H., Yu Y., Yao H. (2020). Application of Coatings
to Alleviate Fireside Corrosion on Heat Transfer Tubes during the
Combustion of Low-Grade Solid Fuels: A Review. Energy Fuels.

[158] Valiente Bermejo M. A., Moya Núñez A., Norling R. (2024). Metal Loss
and Corrosion Attack of FeCrAl Overlay Welds on Evaporator Tube Shields
of a Waste-Fired Power Plant. Mater. Corros.

[159] Zhu M., Yi X., Ma L., Lu J., Zhang H., Lv W. (2026). Corrosion Behavior of HT700 Alloy
in Thermal Storage Chloride Salt
with Sacrificial Anode Protection. Sol. Energy
Mater. Sol. Cells.

[160] Xu X., Wang L., Liu S., Zhang J., Mao X., Fei X., Wu Y., Pu G., Ge F., Chai L., Li B. (2026). Corrosion Behavior
of Laser-Cladding Nickel-Based Coating in High-Temperature
Molten Chloride Salts. Sol. Energy Mater. Sol.
Cells.

[161] Li F., Zhang H., Pei H., Li G., Wen Z., Ai X., Yue Z. (2026). Corrosion-Induced Failure
Mechanism and Life Model
of Low-Cycle Fatigue in Nickel-Based Single Crystal Superalloys. Int. J. Fatigue.

[162] Gada H., Mudgal D., Parvez S., Ahmad B. (2020). Investigation
of high temperature corrosion resistance of Ni25Cr coated and bare
347H SS in actual husk fired boiler atmosphere. Eng. Failure Anal.

[163] Wang G., Liu H., Chen T., Zhang X., Li H., Yu Y., Yao H. (2021). Comparative investigation on thermal
corrosion of alloy coatings in simulated waste incinerator environment. Corros. Sci.

[164] Niemi J., Engblom M., Laurén T., Yrjas P., Lehmusto J., Hupa M., Lindberg D. (2021). Superheater
deposits and corrosion in temperature gradient: Laboratory studies
into effects of flue gas composition, initial deposit structure, and
exposure time. Energy.

[165] Pan P., Zhou W., Zhao Y., Wang Y., Zhang N. (2022). Hot corrosion
behavior of an arc sprayed Fe-based amorphous coating in a simulated
biomass firing environment. Corros. Sci.

[166] Cui J., Zhao H., Yang S., Guo Z., Fu Y., Xu L., Pan H., Xu Y. (2023). Experimental
study on the effect
of fly ash on the combustion characteristics of rice husk in a one-dimensional
furnace. J. Biotechnol. Res.

[167] Guo H., Fan W., Long J., Liu Y. (2023). Influence of surface
condition on the high-temperature corrosion of 12Cr1MoVG, T91, and
TP347H steels in waste-to-energy boilers. Eng.
Failure Anal.

[168] Guo H., Fan W., Liu Y., Long J. (2024). Experimental investigation
on the high-temperature corrosion of 12Cr1MoVG boiler steel in waste-to-energy
plants: Effects of superheater operating temperature and moisture. Process Saf. Environ. Prot.

[169] Maciejczyk A., Maj I., Ciukaj S., Hernik B., Osuch A. (2023). The Influence of Protective Coatings
on High-Temperature Corrosion
under Biomass Ash Deposits. Energies.

[170] Romão E. L., Silva J. M., Luz N. V. S., Conte R. A. (2023). Torrefaction
as a Chlorine Reduction Process of Brazilian Eucalyptus sp. for Use
as Biofuel. BioEnergy Res.

[171] Desiati R. D., Sugiarti E., Hermanto B., Timuda G. E., Izzuddin H., Salsabilla F. A., Anawati A. (2024). Corrosion behavior
of YSZ and YSZ/NiCo coatings on Inconel 625 exposed alkali chlorides. J. Met., Mater. Miner.

[172] Verma, R. ; Kaushal, G. Performance of Thermally Sprayed Inconel Coatings in Erosion-Corrosion Conditions of Biomass-Fired Boilers. In Proceedings of the International Thermal Spray Conference (ITSC 2024), ASM International, Milan, Italy, 2024. DOI: 10.31399/asm.cp.itsc2024p0152.

[173] Bhangria B. K., Sidhu S. S., Ahuja L., Mudgal D. (2024). Effect of
burning bio-mass fuel on the corrosion performance of steels in the
34.5MW boiler. Surf. Rev. Lett.

[174] Agência Nacional de Energia Elétrica (ANEEL). Sistema de Informações de Geração da ANEEL (SIGA); ANEEL, Brasília, 2026. https://dadosabertos.aneel.gov.br/dataset/siga-sistema-de-informacoes-de-geracao-da-aneel. (Accessed 15 July 2026).

[175] Agência Nacional de Energia Elétrica (ANEEL). ANEEL prevê crescimento de 9,1 GW na matriz elétrica brasileira em 2026; ANEEL, Brasília, 2026. https://www.gov.br/aneel/pt-br/assuntos/noticias/2026/aneel-preve-crescimento-de-9-1-gw-na-matriz-eletrica-brasileira-em-2026. (Accessed 15 July 2026).

[176] Associação da Indústria de Cogeração de Energia (COGEN): Capacidade Instalada de Cogeração em Operação Comercial; COGEN, São Paulo, 2026. https://dadosabertos.aneel.gov.br/dataset/usinas-termeletricas-por-tipo. (Accessed 15 July 2026).

[177] Empresa de Pesquisa Energética (EPE). Balanço Energético Nacional 2025: Relatório Síntese—Ano Base 2024; EPE, Rio de Janeiro, 2025. https://www.epe.gov.br/sites-pt/publicacoes-dados-abertos/publicacoes/PublicacoesArquivos/publicacao-885/topico-767/BEN_S%C3%ADntese_2025_PT.pdf. (Accessed 16 July 2026).

[178] Ministério do Trabalho e Emprego. Norma Regulamentadora No. 13 (NR-13): Caldeiras, Vasos de Pressão, Tubulações e Tanques Metálicos de Armazenamento, Ministério do Trabalho e Emprego, Brasília, 2022.

[179] Harun B., Islam M. A. (2026). A Review of Machine Learning-Based
Approaches for Corrosion Rate Prediction. Next
Mater.

[180] Koch, G. ; Varney, J. ; Thompson, N. ; Moghissi, O. ; Gould, M. ; Payer, J. International Measures of Prevention, Application, and Economics of Corrosion Technologies (IMPACT) Study; NACE International: Houston, TX, 2016.

[181] Associação Brasileira de Corrosão (ABRACO). Por que se preocupar com a corrosão?; ABRACO, Rio de Janeiro, 2023. https://abraco.org.br/blog/por-que-se-preocupar-com-a-corrosao/. (Accessed 17 July 2026).

[182] Carraro C. G., Hotza D. (2025). Diagnóstico de resíduos
sólidos industriais em Santa Catarina a partir de manifestos
de transporte de resíduos. Rev. Bras.
Gest. Ambient. Sustentabilidade.

[183] Piske B. E., Lopes F., Utzig J., Wiggers V. R. (2024). Small-scale
Waste Heat Recovery through incineration – a brief review. Renew. Energy Power Qual. J.

